# PRRT2 deficiency induces paroxysmal kinesigenic dyskinesia by regulating synaptic transmission in cerebellum

**DOI:** 10.1038/cr.2017.128

**Published:** 2017-10-20

**Authors:** Guo-He Tan, Yuan-Yuan Liu, Lu Wang, Kui Li, Ze-Qiang Zhang, Hong-Fu Li, Zhong-Fei Yang, Yang Li, Dan Li, Ming-Yue Wu, Chun-Lei Yu, Juan-Juan Long, Ren-Chao Chen, Li-Xi Li, Lu-Ping Yin, Ji-Wei Liu, Xue-Wen Cheng, Qi Shen, You-Sheng Shu, Kenji Sakimura, Lu-Jian Liao, Zhi-Ying Wu, Zhi-Qi Xiong

**Affiliations:** 1 Institute of Neuroscience and State Key Laboratory of Neuroscience, CAS Center for Excellence in Brain Science and Intelligence Technology, Shanghai Institutes for Biological Sciences, Chinese Academy of Sciences, Shanghai 200031, China; 2 Department of Human Anatomy, Guangxi Key Laboratory of Regenerative Medicine & Guangxi Collaborative Innovation Center of Biomedicine, Guangxi Medical University, Nanning, Guangxi 530021, China; 3 University of Chinese Academy of Sciences, Beijing 100049, China; 4 Department of Neurology and Research Center of Neurology, Second Affiliated Hospital, Zhejiang University School of Medicine, Hangzhou, Zhejiang 310009, China; 5 Shanghai Key Laboratory of Regulatory Biology, School of Life Sciences, East China Normal University, Shanghai 200241, China; 6 Institute of Biochemistry and Cell Biology, Shanghai Institutes for Biological Sciences, Chinese Academy of Sciences, Shanghai 200031, China; 7 Department of Cellular Neurobiology, Brain Research Institute, Niigata University, Niigata 951-8585, Japan

**Keywords:** PRRT2, paroxysmal kinesigenic dyskinesia, cerebellum, synaptic transmission

## Abstract

Mutations in the proline-rich transmembrane protein 2 (*PRRT2*) are associated with paroxysmal kinesigenic dyskinesia (PKD) and several other paroxysmal neurological diseases, but the PRRT2 function and pathogenic mechanisms remain largely obscure. Here we show that PRRT2 is a presynaptic protein that interacts with components of the SNARE complex and downregulates its formation. Loss-of-function mutant mice showed PKD-like phenotypes triggered by generalized seizures, hyperthermia, or optogenetic stimulation of the cerebellum. Mutant mice with specific *PRRT2* deletion in cerebellar granule cells (GCs) recapitulate the behavioral phenotypes seen in *Prrt2*-null mice. Furthermore, recording made in cerebellar slices showed that optogenetic stimulation of GCs results in transient elevation followed by suppression of Purkinje cell firing. The anticonvulsant drug carbamazepine used in PKD treatment also relieved PKD-like behaviors in mutant mice. Together, our findings identify PRRT2 as a novel regulator of the SNARE complex and provide a circuit mechanism underlying the PRRT2-related behaviors.

## Introduction

Paroxysmal kinesigenic dyskinesia (PKD) is the most common hereditary paroxysmal movement disorder^1,2^, characterized by attacks of choreic and/or dystonic movements, that are triggered by sudden voluntary movement^2^. The genetic basis for PKD was discovered in 2011 when we successfully identified mutations in the gene *PRRT2* that encodes the proline-rich transmembrane protein 2 in Chinese families with the disease^3^. Since then, there are over 100 reports on *PRRT2* in PKD patients from different ethnic backgrounds^2,4^, and in other paroxysmal disorders including benign familial infantile convulsions (BFIC)^5,6,7,8^ and infantile convulsions with choreoathetosis (ICCA)^9^. However, the biological function of PRRT2 and the pathologic mechanism underlying PKD remain obscure^2,4^.

PRRT2 is an uncharacterized protein that belongs to the PRRT superfamily that contains two predicted trans-membrane domains at its C-terminus, with high homology between human and rodents^3,4,10^. Our previous work has shown that *Prrt2* mRNA is expressed primarily in the central nervous system (CNS)^3^. Previous studies using a yeast two-hybrid system^11^ and biochemical analyses of cultured cell lines^10,12^ have implied the presence of PRRT2 in synapses. PRRT2 was also reported to be associated with a subunit of glutamate receptors^13^. Recently, PRRT2 was shown to interact with the fast Ca^2+^ sensors synaptotagmin 1/2, a protein critical for neurotransmitter release^14^. Although existing data imply the possible involvement of PRRT2 in synaptic regulation, its interacting proteins and subcellular localization remain controversial. Intriguingly, truncated versions of PRRT2 displayed altered subcellular localization in COS7 cells^3^, whereas site-directed mutagenesis of full-length PRRT2 led to an almost complete absence of the protein in cultured neurons^10^. Nevertheless, whether truncation mutation of the *PRRT2* gene in PKD patients results in a loss or gain of function remains unclear.

Despite being one of the most frequent paroxysmal dyskinesia diseases, the neural basis of PKD pathogenesis is largely unknown. In PKD patients, both magnetic resonance imaging (MRI) and autopsies have failed to provide any evidence of significant morphological brain abnormalities^4,15,16^. Dysfunction of the motor cortex or basal ganglia has been considered a possible cause of PKD^4,9,16^. On the basis of other episodic disorders^17^, it has been proposed that PKD might be a channelopathy^18,19^. Yet, multiple genetic studies have ruled out mutation of channel-coding genes as the cause for PKD^4^. The progress of understanding the mechanisms underlying PKD has been impeded by the lack of reliable animal models that mimic the dyskinesia phenotypes of PKD patients and allow invasive mechanistic studies *in vivo*.

In this study, we generated *Prrt2*-mutant mice with a nonsense mutation, as well as several strains of *Prrt2* conditional knockout (cKO) mice. We found reliable induction of dyskinesia attacks resembling human dyskinesia phenotypes in mutant mice and in conditional knockouts that generate mutants in the cerebellum but not forebrain. Our results also showed that PRRT2 is a presynaptic protein that modulates SNARE complex formation. Loss-of-function mutations resulted in abnormality in synaptic transmission at cerebellar parallel fiber (PF)-Purkinje cell (PC) synapses. Importantly, we found that the cerebellum is a key area mediating the function of PRRT2 in controlling movement, as revealed by the observation that conditional knockout of *Prrt2* gene in cerebellar granule cells (GCs) was sufficient to induce dyskinesia attacks. These results provide new insights into the function of PRRT2 in synaptic transmission and movement control, as well as the pathogenic mechanism underlying PKD.

## Results

### Subcellular characterization of endogenous PRRT2

**Figure 1:**
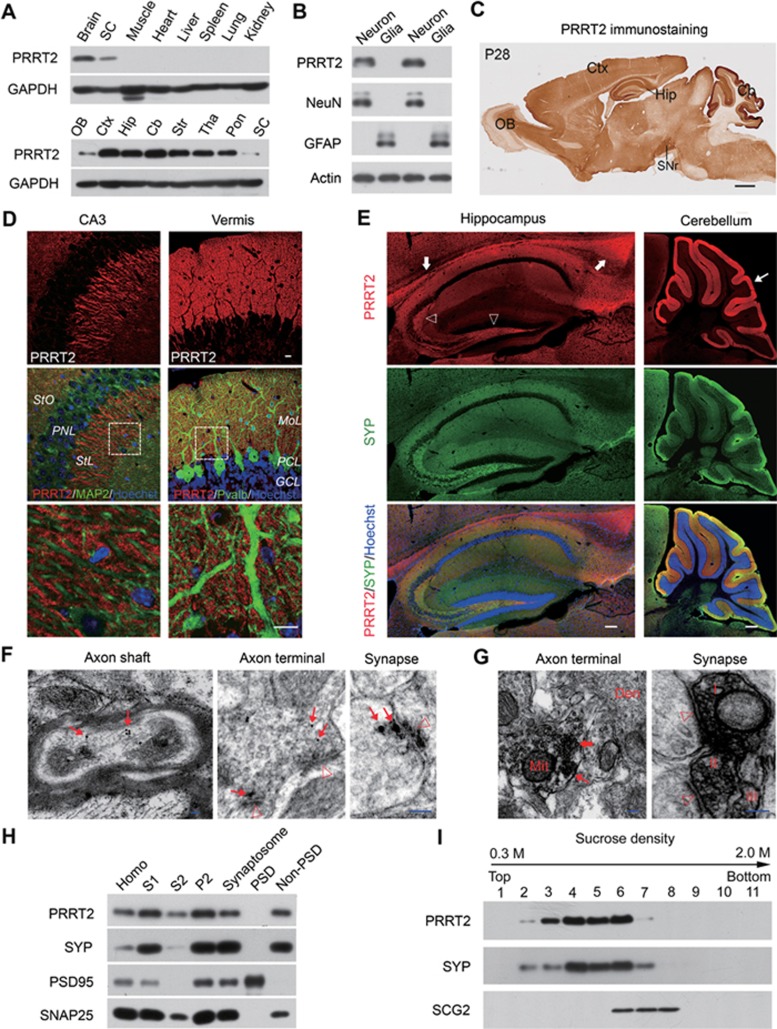
Expression pattern and subcellular localization of PRRT2 in mouse brain. **(A)** Western blotting showing PRRT2 in the nervous system of adult mouse. GAPDH served as an internal control. **(B)** Western blotting showing the expression of PRRT2 in cultured primary neuronal rather than glial cells. NeuN was used as a marker for neurons, while GFAP was for glia cells. **(C)** Representative IHC images of PRRT2 in P28 mouse brain. Scale bar, 800 μm. **(D)** Expression of PRRT2 in mouse brain. MAP2 and Pvalb were used to mark the neuronal dendrites. Boxed areas are showed in high magnification below. Scale bar, 15 μm. **(E)** Co-immunostaining for PRRT2 and presynaptic protein SYP on brain slices. Marked was the intensive expression of PRRT2 in corpus callosum (arrows) in the hippocampal mossy fibers (hollow arrowhead) and the robust expression of PRRT2 in cerebellar molecular layers (thin arrow). Scale bar, 200 μm. **(F)** Immunogold labeling of PRRT2 in neuronal axons and axon terminals. Arrows, PRRT2-positive gold particles; hollow arrowhead, postsynaptic density. Scale bar, 50 nm. **(G)** Pre-embedding immunoperoxidase staining showed that PRRT2 expression was associated with synaptic vesicles in axonal terminals and synapses of the sections that was not counterstained. Thick arrow, labeled small vesicles; thin arrow, labeled presynaptic membrane; hollow arrowheads, postsynaptic density. Roman numerals (I-III) indicate three labeled presynaptic terminals, respectively. Note that there is no labeled signal in postsynaptic membrane and structures. Scale bar, 100 nm. **(H)** Immunoblots of isolated fractions from P30 mouse brain lysates show that PRRT2 did not exist in postsynaptic density. SYP, SNAP25 and PSD95 were used as protein markers to validate different ultrasynaptic compartments. Homo, total brain lysates. **(I)** Immunoblots of equal volume aliquots of the supernatant fractions from mouse brain showed that PRRT2 was present mainly in SYP-containing fractions of small synaptic vesicles. Cb, cerebellum; Ctx, cerebral cortex; Den, dendritic shaft; GCL, granule cell layer; Hip, hippocampus; Mit, mitochondrion; MoL, molecular layer; OB, olfactory bulb; PCL, Purkinje cell layer; Pon, pons; PNL, pyramidal neuronal layer; SC, spinal cord; SNr, substantial nigra; StL, strata lucidum; StO, strata oriens; Str, striatum; Tha, thalamus.

To examine the expression pattern of PRRT2 in different brain regions and subcellular distribution of endogenous PRRT2 protein, we generated a rabbit polyclonal antibody against mouse PRRT2 protein (Supplementary information, Figure S1A). The antibody recognized a single immunoreactive band corresponding to molecular weight of ∼65 kDa in immunoblots of mouse brain (Supplementary information, Figure S1B), larger than the calculated molecular weight of *Prrt2* gene product (∼37 kDa). The specificity of the antibodies was verified by western blotting with lysates from cultured mouse cortical neurons and the results indicate that the immunostained band at 65 kDa indeed represents the endogenous PRRT2. The unexpectedly larger molecular weight was not a result of disulfide bond-mediated dimerization, because treatment with dithiothreitol (DTT) had no effect on its mobility (Supplementary information, Figure S1C). Further analysis showed that PRRT2 protein expression was restricted to the neurons with wide distribution in multiple brain regions (Figure 1A and 1B). Western blotting using mouse brain tissues showed that PRRT2 was highly expressed at perinatal stages and peaked during postnatal weeks 2-4 (Supplementary information, Figure S2A). Immunostaining assays showed that PRRT2 protein is enriched in cerebral cortex, hippocampus, substantia nigra and cerebellum, with prominent expression in neuronal processes rather than cell bodies (Figure 1C and Supplementary information, Figure S2B), resembling the expression pattern of presynaptic proteins (Figure 1D and 1E; Supplementary information, Figure S2C).

Next we performed post-embedding immunogold labeling for electron microscopy (EM). We found that PRRT2 was localized to axon terminals and presynaptic structures (Figure 1F). More interestingly, PRRT2 was observed in the small translucent vesicles (30-80 nm in diameter) in the presynaptic terminals. Furthermore, immunoperoxidase staining also showed PRRT2-immunoreactive vesicles in axonal terminals, with some vesicles located near the presynaptic membrane (Figure 1G). To confirm these results, we performed subcellular fractionation and verified the existence of PRRT2 in the synaptosome rather than PSD fraction (Figure 1H). Furthermore, using subcellular fractionation in a sucrose gradient to separate different vesicle populations in brain lysates, we found that PRRT2 was enriched in small vesicle fractions, but not in large dense-core vesicle fractions (Figure 1I). Together, these results indicate that endogenous PRRT2 is localized to presynaptic membrane and small vesicles, suggesting its potential role in regulating synaptic transmission.

### Truncation mutation depletes PRRT2 protein in the brain

**Figure 2:**
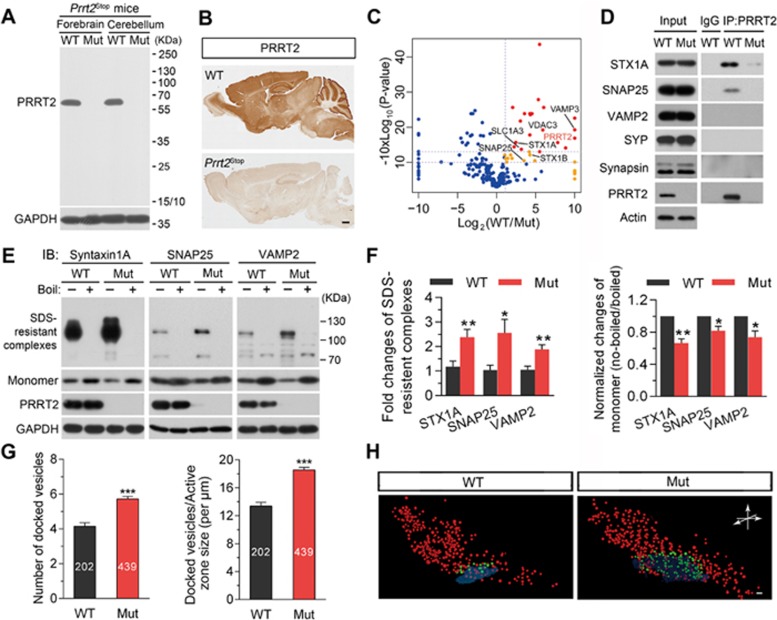
PRRT2 regulates SNARE complex formation and vesicle docking. **(A)** Western blotting did not detect any truncated PRRT2 protein in homozygous *Prrt2^Stop^* mouse brains. The mutant mice have incorporation of two stop codons into their endogenous *Prrt2* gene to mimic the hotspot nonsense mutation at C649 site of human *PRRT2* gene in PKD patients. **(B)** IHC showed that PRRT2 protein is absent in the brain of a *Prrt2^Stop^* mutant mouse. Scale bar, 300 μm. **(C)** Proteomic analysis of PRRT2-interacting proteins using brain lysates from *Prrt2^Stop^* mutants and the WT littermates. Dots: blue, non-specific binders; yellow, medium confident binders; red, highly confident binders. **(D)** Co-IP analysis using mouse brain tissue lysates showed that PRRT2 interacts with STX1A and SNAP25. **(E**, **F)** PRRT2 deficiency resulted in increased formation of SNARE complex within synaptosomes. **E**, representative immunoblots; **F**, quantification of western blot results. Error bars, mean ± SEM. *n* = 5 *per* group; ^*^P< 0.05 and ^**^P< 0.01 vs WT; Student's *t*-test. **(G)** Analysis of vesicle counts in excitatory synapses in cerebellar molecular layer showed significant increase in the number of docked vesicles in mutant mice. Error bars, mean ± SEM. *n* = 202 synapses from 4 WT mice and *n* = 439 synapses from 4 mutant mice. ^***^P < 0.001 vs WT; Student's *t*-test. **(H)** Partial reconstruction of synapses from electron tomography data showed 3D distributions of vesicles in typical asymmetric synapses of cerebellar molecular layer from *Prrt2*-mutant and WT mice. Docked vesicles were labeled in green while others in red. Scale bar, 50 nm. Mut, mutant; WT, wild-type.

To define the function of PRRT2 that endows its essential role in the pathogenesis of PKD, we generated a mouse model carrying mutation that could mimic *PRRT2* mutation found in human PKD patients. The most frequent nonsense mutations of the human *PRRT2* gene are located within a sequence of nine repeated cytosines (c.649 dupC/c.649delC; Supplementary information, Figure S3A). These mutations, which introduce a stop codon *in situ* or downstream of the mutated site, account for the majority of clinical PRRT2-related paroxysmal disorders^1,2,4^. Using a Cas9/CRISPR-mediated gene-targeting strategy, we first generated mice with a *Prrt2^Stop^* mutation, in which two stop codons were introduced into exon 2 of the endogenous murine *Prrt2* gene at a site corresponding to the C649 site of human *PRRT2* (Supplementary information, Figure S3B). DNA sequencing confirmed the correct incorporation of exogenous nucleotides into the target site (Supplementary information, Figure S3C). The resulting mice have normal gross morphology of the brain, with normal foliation and laminated cortical structure (Supplementary information, Figure S3D-S3G) and normal neuronal morphology in the striatum and substantia nigra (Supplementary information, Figure S3H). Notably, the homozygotes expressed normal levels of *Prrt2* transcripts in the brain (Supplementary information, Figure S4A and S4B). Surprisingly, we found complete depletion of PRRT2 protein in these mutant mice (Figure 2A and 2B; Supplementary information, Figure S4C), indicating that *Prrt2^Stop^* is a loss-of-function mutation. To understand what mediates the degradation of truncated PRRT2, we applied MG132 and bafilomycin to inhibit proteasome- or lysosome-mediated protein degradation, respectively. Interestingly, we found that application of MG132 could induce the specific expression of truncated PRRT2 in neuronal cultures from *Prrt2^Stop^* mice (Supplementary information, Figure S4D), indicating that the degradation of truncated PRRT2 was mediated by proteasomes.

### PRRT2 interacts with components of SNARE complex

To better understand the mechanisms underlying the function of PRRT2 in the brain, we searched for its interacting proteins. We carried out co-immunoprecipitation (co-IP) experiments were then carried out with brain tissue lysates, followed by high-resolution mass spectrometry (MS) and label-free quantification (LFQ) as described previously^20^, using *Prrt2^Stop^* mutants as the negative control. We identified a total of 37 proteins that showed significant binding to PRRT2 (Figure 2C). Prominent among these proteins were components of soluble N-ethylmaleimide-sensitive factor attachment protein receptor (SNARE) complex, such as STX1A and SNAP-25 (Figure 2C), suggesting a functional link between PRRT2 and the SNARE complex.

To further confirm the interaction between SNARE components and PRRT2, we performed co-IP assays followed by western blotting against synaptic proteins. We found that anti-PRRT2 antibodies efficiently and specifically co-immunoprecipitated STX1A and SNAP-25, but not the other SNARE complex component synaptobrevin (also referred to as VAMP), as well as other presynaptic proteins such as SYP and SYS1 (Figure 2D). In contrast, we failed to co-immunoprecipitate STX1A and SNAP-25 in parallel co-IP experiments using brain lysates of *Prrt2^Stop^* mice, or brain lysates of WT mice pre-incubated with a peptide synthesized according to the epitope of PRRT2 antibody, indicating the specificity of the PRRT2 interaction with these synaptic proteins.

### PRRT2 inhibits SNARE complex formation

We then attempted to examine the significance of PRRT2 interaction with components of the SNARE complex. Since assembled SNARE complexes are resistant to 1% SDS lysis buffer at room temperature but can be dissociated into monomeric SNARE proteins at 100 °C^21,22^, we compared the amount of assembled SNARE complexes using synaptosome samples from WT and *Prrt2^Stop^* mice at room temperature. Interestingly, the amount of assembled SNARE complex was significantly increased in the lysates from the mutant mouse brain, accompanied by a reduction in the amount of monomeric STX1A, SNAP-25 and VAMP2 (Figure 2E and 2F; Supplementary information, Figure S5A-S5C). In contrast, PRRT2 inhibited the formation of SNARE complexes in HEK293T cells that were transfected with plasmids encoding exogenous SNARE complex components (Supplementary information, Figure S5D). These results indicate that interaction of PRRT2 with SNARE components inhibits formation of the SNARE complex, which is known to be critical for synaptic vesicle docking and exocytosis^22^.

### PRRT2 level affects synaptic vesicle docking

We further examined whether PRRT2 deficiency could influence synaptic vesicle docking by quantitative ultra-structural analysis of synaptic vesicle distribution in the presynaptic nerve terminal. We focused on asymmetric excitatory synapses in the cerebellar molecular layer, where presynaptic PRRT2 was highly enriched (Figure 1C-1E). We first compared cerebellar sections of 4-week-old *Prrt2^Stop^* mutant mice with those of their WT littermates, using transmission EM (Supplementary information, Figure S6A-S6C), and found no significant difference in the density of synapses and presynaptic active zone length between these two groups (Supplementary information, Figure S6D). However, we observed a significantly higher number of docked vesicles in sections from *Prrt2*-mutant mice (Figure 2G; Supplementary information, Figure S6B and S6C; docked vesicles per μm of active zone, 13.3 ± 0.4 and 18.4 ± 0.4 (*P* < 0.001), respectively, for WT and mutant mice).

Furthermore, 3D reconstruction of serial EM images of 5-nm sections obtained by focused ion beam/scanning EM (FIB/SEM) showed a higher number of docked synaptic vesicles in the presynaptic membrane in PRRT2-deficient synapses in comparison to WT controls (Figure 2H and Supplementary information, Movie S1), consistent with transmission EM results. Taken together, these data suggest that the loss of PRRT2 leads to an increase of synaptic vesicle docking.

### PRRT2 downregulates short-term facilitation in cerebellum

**Figure 3:**
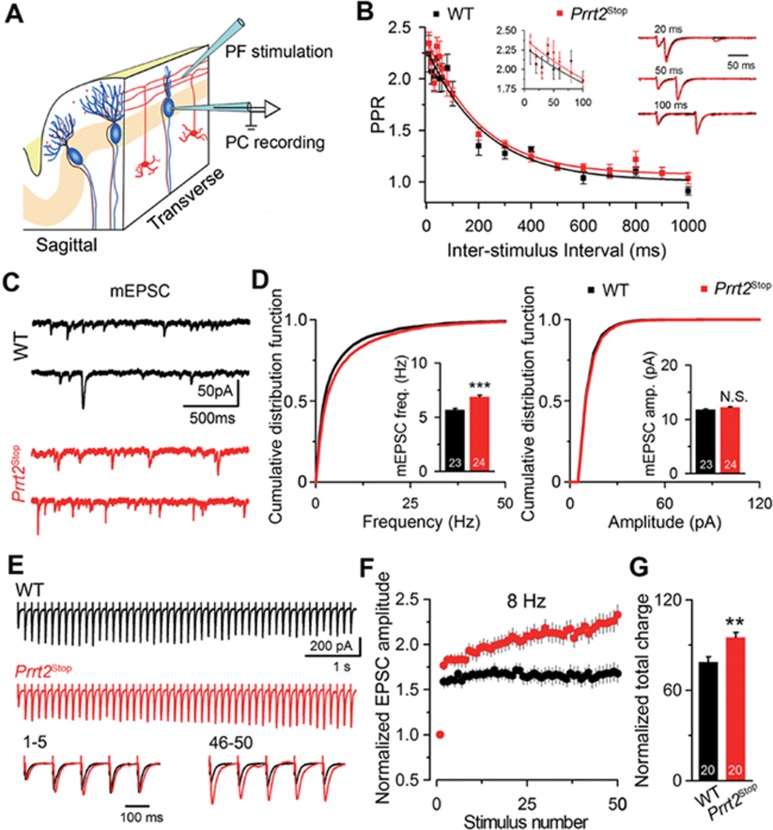
PRRT2 deficiency increased short-term facilitation of PF-PC synapses in cerebellum. **(A)** Illustration of the electrophysiological recording configuration. Electrical stimulation was delivered onto GC-derived PFs and whole-cell voltage-clamp recording was performed on the somata of PCs. **(B)** PPR at intervals from 10 to 1 000 ms in *Prrt2^Stop^* mice and WT littermates. Mono-exponential fits to the data were shown as solid lines. Left inset shows PPR values at very short ISIs (10-100 ms) on an expanded timescale. Right inset shows examples of paired-pulse traces at 20, 50, 100 ms intervals, scaled to the first EPSC in the pair for each interval. Error bars, mean ± SEM. WT, *n* = 7; mutant, *n* = 6. **(C)** Representative recording of mEPSCs in cerebellar slices from *Prrt2^Stop^* mice and their WT littermates. **(D)** Cumulative frequency or amplitude distribution curves of mEPSCs calculated for WT (black) and *Prrt2^Stop^* (red) mice. The sample size is indicated in the bottom of each bar. All cumulative curves and mean values were obtained from 200 events recorded from each cell. WT, *n* = 23; mutant, *n* = 24. Error bars, mean ± SEM. ^*^P< 0.05 and ^***^P< 0.001 vs WT; Student's *t*-test. **(E)** Representative traces of PF-EPSCs evoked by a stimulus train of 50 pulses at 8 Hz in WT (black) and *Prrt2^Stop^* (red) mice. Bottom, the traces of the 1^st^-5^th^(left) and 46^th^-50^th^ (right) PF-EPSCs were peak scaled to the amplitude of the 1^st^ EPSCs. **(F)** Normalized plot of EPSC amplitudes of each spike during trains of stimulations at 8 Hz. *X*-axis, stimulation numbers. WT, black; mutant, red. *n* = 20 *per* group. **(G)** Total charge transferred (normalized to that of the 1^st^ EPSC) during the trains of 50 pulses at 8 Hz. Error bars, mean ± SEM. ^**^P< 0.01 vs WT; Student's *t*-test. The sample size is indicated in the bottom of each column. NS, not significant; PC, Purkinje cells; PF, parallel fibers; PPR, paired-pulse ratio.

Given the role of the SNARE complex in synaptic transmission^22^, we next examined whether the loss of PRRT2 affects the function of excitatory synapses in the cerebellar molecular layer^23^ (Figure 3A). Whole-cell recording from PCs showed that there was no significant difference in the paired-pulse ratio (Figure 3B) and in the mean amplitude of miniature excitatory postsynaptic currents (mEPSC) at PF-PC synapses between the *Prrt2^Stop^* mutant and WT mice (Figure 3C and 3D), suggesting that PRRT2 does not play a role in regulating the transmitter release probability^24^ or postsynaptic responses to the released transmitter at these synapses. However, the mean mEPSC frequency was significantly higher in *Prrt2^Stop^* mutants than in WT mice (*P* < 0.001). We further found that *Prrt2^Stop^* PF-PC synapses exhibited a higher level of synaptic facilitation than that of WT synapses in response to a train of stimuli, with increasing amplitudes of evoked EPSCs triggered by later stimuli (Figure 3E and 3F). This was also shown by the higher total charge associated with all EPSCs induced by the train (*P* = 0.003; Figure 3G). Together, these data indicate that PRRT2 deficiency resulted in facilitated synaptic transmission at PF-PC synapses when these synapses were repetitively activated.

### Behavioral characterization of PRRT2-deficient mice

Because the clinical criteria used to diagnose PKD are based on behavioral symptoms rather than molecular or anatomical indicators, we then examined behavioral phenotypes of *Prrt2^Stop^* mice. We found that *Prrt2^Stop^* mutant mice exhibited largely normal gait (Supplementary information, Figure S7A), locomotion (Supplementary information, Figure S7B), anxiety-related responses (Supplementary information, Figure S7C-S7E) and muscle strength (Supplementary information, Figure S7F-S7H). Moreover, we found no obvious deficits in sensory functions (Supplementary information, Figure S7I-S7K). However, we observed that *Prrt2^Stop^* mice displayed deficits in both beam-walking test and accelerating rotarod test, two measures of motor coordination and balance (Supplementary information, Figure S8A and S8B). We also used conditional *Prrt2*-knockout mice in which PRRT2 was selectively eliminated from the CNS, using a Cre/*LoxP* strategy (referred to as *Nestin-Cre;Prrt2^−/−^*; Supplementary information, Figure S9A and S9B). Although we detected the presence of residual transcripts in homozygous mutant mice (none containing exon 2; Supplementary information, Figure S9C), no residual truncated proteins with low molecular weights were detected in the brain (Supplementary information, Figure S9D), suggesting complete depletion of PRRT2 protein. Like *Prrt2^Stop^* mice, *Nestin-Cre;Prrt2^−/−^* mice developed normally (Supplementary information, Figure S9E-S9G) and showed poor performance in motor coordination functions (Supplementary information, Figure S9H and S9I). Together, our findings provide a link between PRRT2 deficiency and impairment of motor coordination.

### PRRT2-deficient mice exhibit induced PKD-like behaviors

PKD is characterized by dystonic and/or choreic movements^1,2,3^. Although mutant mice are unlikely to match precisely the clinical symptoms of PKD, we did observe spontaneous dyskinesia attacks in some PRRT2-deficient mice under natural conditions. Each mouse exhibited a different subset of dyskinetic or dystonic behaviors (Supplementary information, Movie S2). The spontaneous dyskinesia attacks were observed at a rather low frequency. We thus used multiple strategies to induce dyskinesia attacks in the mutant mice. Most cases of human PKD are initiated by defined inducers, such as the sudden initiation of movement, startle and even passive manipulation of muscles^3,4,25^. However, we failed to induce dyskinesia attacks by using stimulation methods^26,27,28^ including stressors, electric foot shock, cocaine, caffeine, ethanol, morphine, quinpirole and running.

**Figure 4:**
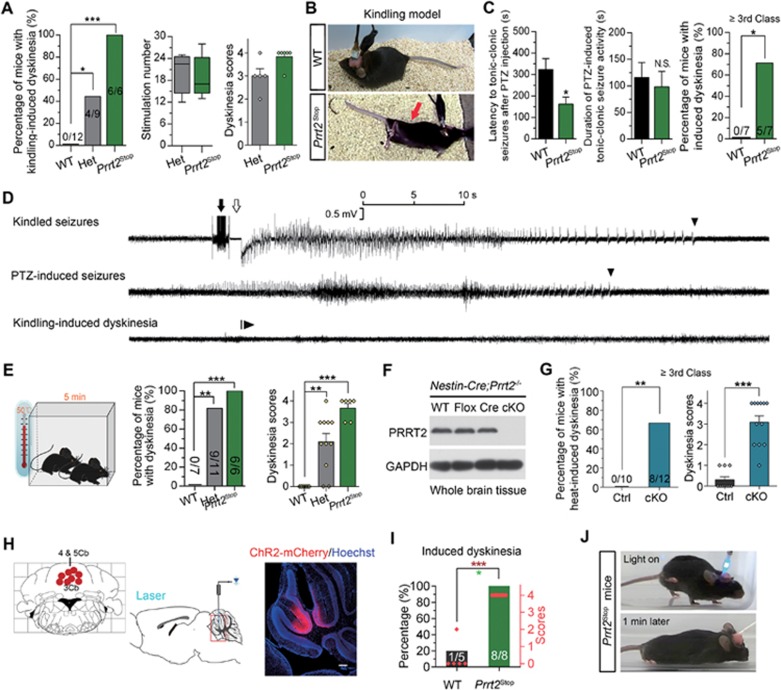
Induced dyskinesia in *Prrt2*-deficient mice. **(A)** *Prrt2^Stop^* mice manifested profound paroxysmal dyskinesia after kindling-induced generalized seizures. Left panel, incidence of generalized seizure-induced dyskinesia; the number of mice in each group is shown in the columns. Middle panel, box-and-whisker plot shows the range of stimulations required to evoke the seizure-induced dyskinesia in mutant mice. Right panel, dyskinesia scores of mutant mice after kindling-induced seizure activity; individual symbols indicate all the cases in each group. ^*^P< 0.05 and ^***^P < 0.001 vs WT; Fisher's exact test. **(B)** Representative images of dyskinesia attack in a *Prrt2^Stop^* mouse in kindling model. Arrow indicates a rigor and hunched posture. **(C)** Paroxysmal dyskinesia triggered by PTZ-induced generalized seizures in *Prrt2^Stop^* mice. Cases are indicated in the column of each group. ^*^P < 0.05 vs WT; Fisher's exact test. **(D)** Representative EEG recordings of *Prrt2^Stop^* mice during kindled seizures (top), PTZ-evoked seizures (middle) and induced dyskinesia (bottom). Filled arrow, application of electrical stimulation in kindling; open arrow, post-stimulation refractory period; arrowheads, termination point of electrographic seizures. Note there were no obvious abnormalities in EEG recordings during occurrence of induced dyskinesia. **(E)** 5-min exposure to 50 °C ambient temperature induced paroxysmal dyskinesia in *Prrt2^Stop^* mice. ^**^P < 0.01 and ^***^P < 0.001 vs WT; middle panel, Fisher's exact test; right panel, one-way ANOVA with *post hoc* Dunnett's test. **(F**, **G)** Exposure to 50 °C for 5 min induced dyskinesia in *Nestin-Cre;Prrt2^−/−^* mice. Individual symbols indicate all the cases in each group. *Nestin-Cre* and *Prrt2^loxP/loxP^* littermates expressing PRRT2 normally were pooled as controls. ^*^P < 0.05, ^**^P < 0.01 and ^***^P < 0.001 vs control littermates; Fisher's exact test or Student's *t*-test. **(H)** Schematic diagram (left and middle) and representative image of ChR2-mCherry expression (right) for optogenetic manipulation in *Prrt2^Stop^* mice. Distribution of the actually implanted sites was indicated using red bulb individually. **(I)** Optical stimulation in cerebellar vermis sufficiently induced intensive paroxysmal dyskinesia in *Prrt2^Stop^* mice, but not in WT mice. Individual symbols indicate all the cases in each group. ^*^P < 0.05 and ^***^P < 0.001 vs WT; Student's *t*-test. **(J)** Representative images of optogenetic stimulation-induced dyskinesia in *Prrt2^Stop^* mice. Het, heterozygote.

Finally, we resorted to kindling stimulation used in epilepsy models, in which repeated administration of an initially subconvulsive electric stimulus results in progressive intensification of seizures, culminating in generalized seizures^29^. We observed that dyskinesia attacks occurred in all *Prrt2^Stop^* homozygotes (6/6) and about half of the heterozygotes (4/9), but none (0/12) of the WT littermates (Figure 4A and 4B; Supplementary information, Movie S3). Attacks in these mice were often preceded by strong behavioral seizures with an intensity of over class 3. The onset of dyskinesia had a latency of 15 s to 5 min after seizure attack, lasting more than 30 min with a typical paroxysmal pattern. Attacks in the homozygotes usually began with a combination of involuntary choreiform movements that rapidly spread from the head to the limbs, and were followed by dystonic postures of the trunk, limbs and the tail. The heterozygotes predominantly showed choreoathetosis such as drastic tremors in the forelimbs and face accompanied by sialorrhea of a relatively low degree and a shorter duration compared to the homozygotes. Based on previous studies of mouse dyskinesia, we set a disability scale to determine the overall functional impact of the abnormal movements^25^ (Materials and Methods). After dyskinesia attacks, the mice recovered to normal locomotion.

To determine whether seizure-induced dyskinesia is a general phenomenon in mutant mice, we turned to another widely used epilepsy model, using pentylenetetrazol (PTZ; a non-competitive GABAa receptor antagonist) to evoke robust seizures in the mice, with a short latency of several minutes^30^. Intraperitoneal injection of PTZ induced class-5 generalized seizures with a shorter latency in *Prrt2^Stop^* mice than WT mice (Figure 4C and 4D). As observed in the kindling model, PTZ-induced seizures also caused dyskinesia attack after generalized seizures in most of the homozygous mutants (5/7), but not in WT mice (Figure 4C and Supplementary information, Movie S4). Thus, the above assays could be used to study experimentally induced dyskinesia.

*PRRT2* mutations have also been reported in patients with febrile seizures^8^. This suggested that *PRRT2*-deficient mice might have similarly increased sensitivity to hyperthermia. To test this idea, we placed *Prrt2^Stop^* mice and their controls in boxes with a set ambient temperature. Strikingly, we found that all homozygous mutants (6/6) but none of the WT controls (0/7) developed dyskinesia attacks 5 min after being exposed to a 50 °C environment (Figure 4E and Supplementary information, Movie S5). Most heterozygous *Prrt2^Stop^* mice also exhibited dyskinesia (9/11) (Figure 4E). Heat-induced dyskinesia also occurred consistently in *Nestin-Cre;Prrt2^−/−^* mice (Figure 4F and 4G).

In view of the fact that paroxysmal movement disorders do not have typical epileptic characteristics and ictal/inter-ictal EEGs are normal in the vast majority of cases^3,4,16^, we performed EEG recording in animal models to discriminate dyskinesia from epileptic seizures. There were no epileptiform discharges or obvious EEG abnormalities during experimentally induced dyskinesia (Figure 4D and Supplementary information, Movie S6), in accord with observations in human patients^16,31^. Recent clinical data have shown that paroxysmal diseases associated with PRRT2 mutations respond well to some anti-convulsant drugs, such as carbamazepine (CBZ)^32^. We therefore treated *Prrt2^Stop^* mice with CBZ after PTZ-induced generalized seizures. Strikingly, administration of CBZ significantly alleviated dyskinesia phenotypes in the mutant mice (Supplementary information, Figure S10A; dyskinesia score from 3.7 ± 0.3 to 1.8 ± 0.6, *P* = 0.029, for 15 min later; from 3.3 ± 0.4 to 1.2 ± 0.4, *P* = 0.027, for 30 min later; *n* = 6), further validating our mouse models.

### Cerebellar activation is sufficient for experimentally induced PKD

Aberrant cerebellar activity has been implicated in the generation of involuntary dystonia movements^33,34^. To test this possibility in *Prrt2^Stop^* mutant mice, we performed optogenetic manipulations to directly stimulate their cerebellum. Adeno-associated virus (AAV) vectors expressing green fluorescent protein (GFP) and channelrhodopsin-2 (ChR2) were stereotactically delivered into the cerebellar vermis of *Prrt2^Stop^* mice (Figure 4H). Interestingly, *in vivo* light stimulation of ChR2 in infected cerebellar neurons was sufficient to induce dyskinesia in *Prrt2^Stop^* mice (Figure 4I and 4J). These findings suggest that local cerebellar activation is sufficient for involuntary dyskinesia movements in *Prrt2*-deficient mice.

### Cerebellar GC-specific deletion of PRRT2 is sufficient to induce dyskinesia

**Figure 5:**
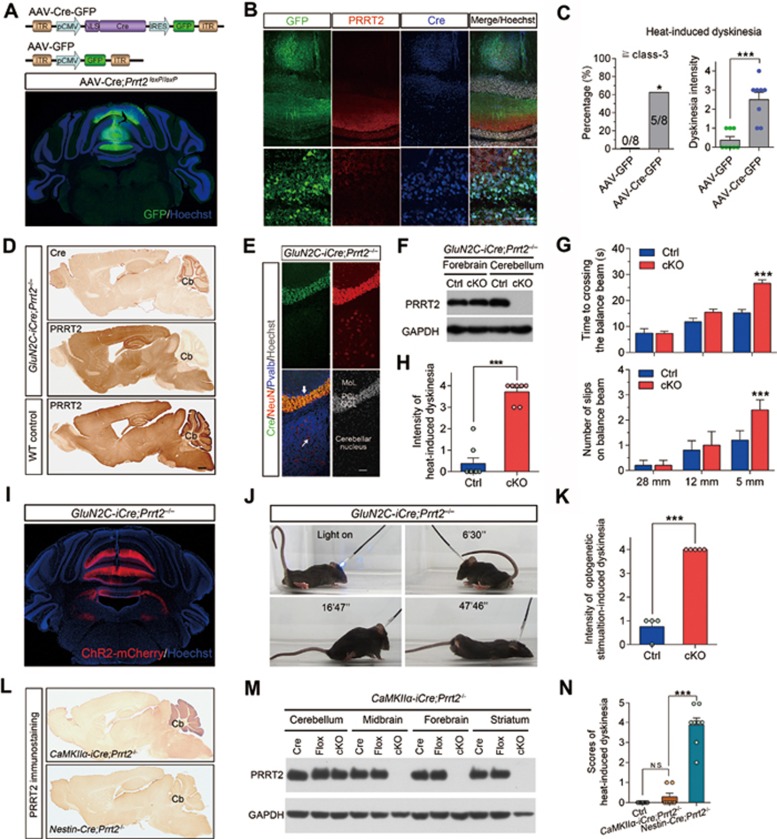
Cerebellum is a key area mediating PRRT2-related dyskinesia. **(A)** Schematic diagrams of AAV construct (upper panel) and representative image of AAV injection into the vermis of cerebellum in *Prrt2^loxP^*^/^*^loxP^* mice (lower panel). **(B)** Immunostaining results showing that AAV-mediated Cre expression ablated PRRT2 protein in the infected neurons. Scale bar, 50 μm. **(C)** Incidence and scores of paroxysmal dyskinesia in AAV-*Cre;Prrt2^−/−^* and control mice after exposure to 50 °C ambient temperature. Numbers (upper panel) or symbols (lower panel) in the column indicate the cases in each group. ^*^P < 0.05 and ^***^P < 0.001 vs GFP controls; upper panel, Fisher's exact test; lower panel, Student's *t*-test. **(D)** Generation of *GluN2C-iCre;Prrt2^−/−^* mice in which PRRT2 was ablated selectively in cerebellar GCs revealed by IHC staining. Shown are two neighboring sagittal sections of the homozygote brain stained for Cre and PRRT2, respectively. **(E)** Multiple-label immunostaining showed specific Cre expression in *GluN2C-iCre;Prrt2^−/−^* mouse cerebellum. Arrow indicates a typical Purkinje cell that did not express Cre. Thin arrow indicates a neuron in deep nucleus of cerebellum without Cre expression. Scale bar, 100 μm. **(F)** Western blot analysis of PRRT2 expression in brains from *GluN2C-iCre;Prrt2^−/−^* mice and the control littermates. **(G)** Analysis of *GluN2C-iCre;Prrt2^−/−^* mice in beam balance tests. *GluN2C-iCre* and *Prrt2^loxP/loxP^* littermates carrying both alleles of *Prrt2* gene were pooled as controls. WT, *n* = 17; mutant, *n* = 14. Error bars, mean ± SEM. ^***^P < 0.001 vs control; two-way ANOVA with *post hoc* Bonferroni's test. **(H)** Incidence and scores of heat-induced paroxysmal dyskinesia of *GluN2C-iCre;Prrt2^−/−^* mice. Numbers (left panel) or symbols (right panel) in the column indicate the cases in each group. ^**^P< 0.01 and ^***^P< 0.001 vs control; Fisher's exact test or Student's *t*-test. **(I)** Representative images of ChR2-mCherry expression in *GluN2C-iCre;Prrt2^−/−^* mouse cerebellum after AAV injection. **(J)** Representative images of optogenetic stimulation-induced dyskinesia in a *GluN2C-iCre;Prrt2^−/−^* mouse at the indicated time points. **(K)** Statistical analysis of scores of optogenetic stimulation-induced dyskinesia in *GluN2C-iCre;Prrt2^−/−^* mice. Individual symbols indicate all the cases in each group. Error bars, mean ± SEM. ^*^P < 0.05 vs control; Student's *t*-test. **(L)** Generation of *CaMKIIα-iCre;Prrt2^−/−^* mice. IHC results showed normal expression of PRRT2 in the cerebellum of *CaMKIIα-iCre;Prrt2^−/−^* mice. Brain sections from *Nestin-Cre;Prrt2^−/−^* mice were used as the control. **(M)** Western blot analysis of PRRT2 expression in distinct brain regions of both *CaMKIIα-iCre;Prrt2^−/−^* mice and their littermate controls. **(N)** Heat-induced dyskinesia scores of *CaMKIIα-iCre;Prrt2^−/−^* mice. Individual symbols indicate all the cases in each group. Both *Nestin-Cre;Prrt2^−/−^* and WT mice were used as controls.

As the cerebellum expresses a high level of PRRT2 and plays an important role in regulating movements^35^, we examined the role of the cerebellum in the mouse dyskinesia model. We injected AAV-Cre-GFP into the cerebellum of *Prrt2^loxP^*^/^*^loxP^* mice to induce local ablation of the *Prrt2* gene (*AAV-Cre;Prrt2^−/−^*; Figure 5A). Immunostaining showed that PRRT2 was selectively ablated in the infected neurons (Figure 5B). Notably, hyperthermia-induced dyskinesia occurred in all mice harboring the AAV-Cre-mediated PRRT2 deletion (Figure 5C). The dyskinesia scores were significantly higher in *AAV-Cre;Prrt2*^−/^^−^ mice (2.5 ± 0.4, *n* = 8; *P* = 0.0002 vs control) than in the control group with AAV-GFP injection (0.4 ± 0.2, *n* = 8). Thus, local ablation of PRRT2 in the vermis of the cerebellum increased susceptibility to hyperthermia-induced dyskinesia, strongly supporting an important role of cerebellar PRRT2 in movement disorders.

To further elucidate the circuit mechanism underlying PRRT2-related dyskinesia in the cerebellum, we generated a mouse line (*GluN2C-iCre;Prrt2^−/−^*) with ablation of PRRT2 in cerebellar GCs (Figure 5D-5F), but not in other cerebellar cells, by crossing *Prrt2^loxP^*^/^*^loxP^* mice with *GluN2C-iCre* mice^35^. Behavioral analysis showed that the resulting *GluN2C-iCre;Prrt2^−/−^* mice exhibited defects in beam walking (Figure 5G), and dyskinesia 5 min after exposure to a 50 °C environment (Figure 5H). In addition, similar to *Prrt2^Stop^* mice, the *GluN2C-iCre;Prrt2^−/−^* mice also showed facilitated synaptic transmission at PF-PC synapses, as indicated by a gradual increase in the amplitude of evoked EPSCs in response to a train of stimuli (Supplementary information, Figure S11A-S11E).

We next stereotactically delivered AAV-expressing ChR2 into the cerebellar vermis of *GluN2C-iCre;Prrt2^−/−^* mice (Figure 5I). Intriguingly, *in vivo* light stimulation of ChR2 in AAV-infected cerebellar neurons induces dyskinesia in *GluN2C-iCre;Prrt2^−/−^* mice (Figure 5J and 5K; Supplementary information, Movie S7), indicating that a PRRT2 function within cerebellar GCs is critical for normal movement control.

We also generated *CaMKIIα-iCre;Prrt2^−/−^* mice, in which *Prrt2* was locally ablated in the forebrain, including motor cortex and stratium (Figure 5L and 5M), but normally expressed in cerebellum. In contrast to the intense dyskinesia found in all *GluN2C-iCre;Prrt2^−/−^* mice, hyperthermia hardly induced dyskinesia attacks in *CaMKIIα-iCre;Prrt2^−/−^* mice (Figure 5N). Taken together, these results confirm that cerebellum is a key region for PRRT2-related dyskinesia.

### Abnormal PC firing in PRRT2-deficient cerebellum

**Figure 6:**
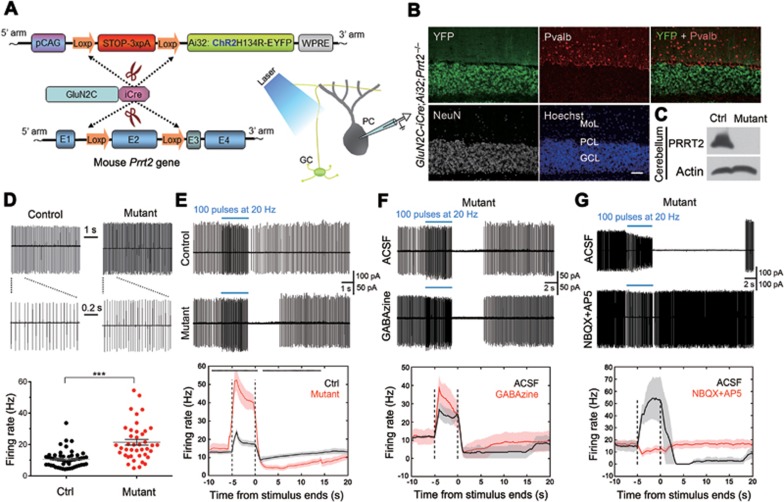
Abnormal PC firing of *Prrt2*-deficient mice. **(A)** Schematic diagrams for the generation of *GluN2C-iCre;Ai32;Prrt2^−/−^* mice. **(B)** IHC results showed specific expression of the ChR2-YFP fusion proteins in all GCs (white) in cerebellar cortex, but not in Pvalb-positive PCs and other GABAergic interneurons (red). Scale bar, 400 μm. **(C)** Western blotting shows complete depletion of PRRT2 protein in *GluN2C-iCre;Ai32;Prrt2^−/−^* mouse cerebellum (*−/−*). *GluN2C-iCre;Ai32;Prrt2^+/+^* mice were used as the control (*+/+*). **(D)** Spontaneous firing activity recorded from PCs on *GluN2C-iCre;Ai32;Prrt2^+/+^* and *GluN2C-iCre;Ai32;Prrt2^−/−^* mouse cerebellar slices. Upper: representative traces. The first 1-s segments indicated by lines in the upper panels are shown at higher magnification below. Lower: statistical analysis of average firing rate. Each mark in the scatter plots represents individual average firing properties. Bars indicate mean ± SEM. **(E)** Spontaneous firing activity of PCs after optogenetic stimulation (blue bar). Upper: representative traces. Lower: summary of spontaneous firing frequency. Black, *n* = 40 cells from 8 *GluN2C-iCre;Ai32;Prrt2^+/+^* mice. Red, *n* = 30 cells from 7 *GluN2C-iCre;Ai32;Prrt2^−/−^* mice. Black asterisks on the top indicate statistical significance at individual time points. **(F)** Cell-attached recording of PCs in the absence or presence of GABAa receptor blocker GABAzine. **(G)** Effect of glutamate receptor blocker NBQX and AP5 on PC firing activity after optogenetic stimulation.

To establish the link between synaptic changes at GC-PC synapses and cerebellar circuit function, we generated a mouse line in which ChR2 was specifically expressed and PRRT2 was specifically ablated in cerebellar GCs (Figure 6A-6C), by crossing Ai32 transgenic mice^36^ with *GluN2C-iCre;Prrt2^−/−^* mice. Using cell-attached recordings of PCs under optogenetic stimulation of GCs in acute transverse cerebellar slices, we examined the effect of the PRRT2 deletion on PC firing. In both mice lacking PRRT2 (*GluN2C-iCre;Ai32*^+/−^;*Prrt2^−/−^*) or expressing PRRT2 (*GluN2C-iCre;Ai32*^+/−^;*Prrt2*^+/+^), PCs exhibited tonic firing in majority of cells (+/+, 75.8%; −/−, 73.8%). However, the tonic PC firing frequency was much higher in slices with PRRT2 deleted in GCs (+/+, 10.7 ± 0.7 Hz, *n* = 53 cells; −/−, 21.5 ± 1.9 Hz, *n* = 41 cells; *P* < 0.0001, Student's *t*-test; Figure 6D). We found that stimulating GCs with blue light flashes induced a marked increase of PC firing; *Prrt2*-deficient mice (*n* = 30 cells, 9 mice) showed a much higher increase than control mice (*n* = 40 cells, 8 mice; Figure 6E). Surprisingly, PC firing was greatly suppressed following the light stimulation for up to 20 s for *Prrt2*-deficient mice, whereas only a slight suppression was observed for the controls. Specifically, post-stimulus PC firing was totally eliminated for a few seconds in 86% of PCs in *Prrt2*-deficient mice, but only in 9% of PCs in control mice.

PC firing is bi-directionally modulated through excitatory synapses made by PFs and inhibitory synapses made by cerebellar interneurons^37,38^. To determine the cause of elevated stimulus-induced firing and post-stimulation suppression of PCs upon PRRT2 deletion, we applied specific blockers of GABAa and glutamate receptors. Perfusion of Gabazine had no significant effect on the elevated light-induced firing of PCs (Figure 6F). On the other hand, treatment with NBQX and AP5 significantly eliminated both stimulus-induced elevation and post-stimulation suppression of PC firing (Figure 6G). These results support the notion that the abnormal firing of PCs from *Prrt2*-deficient mice during and following optogenetic stimulation was due to facilitated transmission at synapses made by GCs and interneurons on PCs. Taken together, these results indicate that specific ablation of PRRT2 in GCs can induce abnormal spiking activity of PCs, accounting for the dyskinesia attacks.

## Discussion

Since 2011, genetic linkage studies have implicated *PRRT2* as a causative gene for PKD, BFIC, ICCA and other related neurological disorders^3,4,5^. However, the physiological function of the poorly characterized PRRT2 protein has been unclear^39^. This has been in part due to the unavailability of specific commercial antibodies against the protein. The specific anti-PRRT2 antibodies generated in this work identified a protein from both mouse brain and cultured neurons migrating with the molecular weight of ∼65 kDa in western blotting. We suspect that the apparent molecular weight of PRRT2 detected by immunoblotting is greater than its calculated molecular mass because of its proline-rich domain. Similarly, MeCP2, which contains a proline-rich domain, also shows abnormal migration in gels^40^. Here, endogenous PRRT2 protein was shown to be selectively enriched in the CNS and its expression peaked at postnatal stages, in parallel to the mRNA expression pattern revealed by our previous work^3^. Our results further reveal selective localization of endogenous PRRT2 protein in presynaptic terminals and its regulation in SNARE complex formation. However, we did not detect interaction of PRRT2 with synaptotagmin, as reported by Benfenati's lab^14^. In addition, *Prrt2* mutation results in an increased amount of the SNARE complex and docked vesicles, suggesting a potential role in regulation of synaptic transmission.

Whether loss or gain of function of PRRT2 underlies the symptoms of PKD has been controversial^3^. Among the identified PRRT2 mutations, most are truncation mutations^2,3,4^ that theoretically produce truncated PRRT2 proteins lacking the transmembrane domains required to anchor the protein to the membrane^3,10^. It has been hypothesized that soluble mutant PRRT2 may produce a dominant negative effect, similar to some soluble membrane receptors^41^. Here, we provide *in vivo* evidence that truncation mutation of the *Prrt2* gene in mice results in complete loss of PRRT2 expression in brain that was mediated by its degradation through the proteasome. This is a similar consequence to the knockout of this gene, excluding the possibility of gain-of-function mechanisms of truncated PRRT2 in the pathogenesis of the PKD-ICCA-BFIC disease spectrum.

Spontaneous dyskinesia attacks occur in some *Prrt2*-deficient mice under natural conditions, consisting of a collection of various abnormal movements. These phenotypes are distinct from the findings of Benfenati's lab^42^ and represent different subsets of dyskinetic or dystonic behaviors^2,4,38,39,43^. For the first time, we have generated several experimental mouse models mimicking PRRT2-related dyskinesia manifested in human patients, including thermal elevation, kindling or PTZ-induced seizures, and optogenetic stimulation (Figure 4). The precise anatomic substrates or pathophysiological networks associated with PKD remain unclear so far. Many studies prior to ours have proposed that the alteration of the basal ganglia-thalamocortical circuit may contribute to the pathogenesis of PRRT2-related PKD^44,45^. Both our histological and biochemical experiments have shown that PRRT2 is highly expressed in cerebellar GCs (Figure 5D-5G, 5L-5N), suggesting a potentially important role of cerebellum in PRRT2-related movement disorders. Significantly, *Prrt2*^loxP/loxP^ mice with AAV-Cre-mediated knockout of PRRT2 in the midline cerebellum displayed hyperthermia-induced dyskinesia (Figure 5A-5C). In addition, *GluN2C-iCre;Prrt2^−/−^* mice, with selective deletion of PRRT2 in cerebellar GCs also generate optogenetic-induced dyskinesia phenotypes similar to *Prrt2^Stop^* mutant mice (Figure 5I-5J). These results indicate activation of cerebellum is sufficient to induce dyskinesia attacks in *Prrt2*-deficient mice. Meanwhile, *CaMKII-iCre;Prrt2^−/−^* mice with ablation of PRRT2 in forebrain could not be induced to develop dyskinesia by optogenetic stimulation of the cerebellum, PTZ injection and hyperthemia. Together, these results identify the cerebellum as a key region for PRRT2-related dyskinesia.

Cerebellar GCs account for a majority of neurons in the cerebellar cortex. These cells send PFs up through the PC layer into the molecular layer where they branch out and spread through PC dendritic arbors and cerebellar cortical interneurons^23,38^. Bidirectional modulation of PC firing frequency is possibly through excitation mediated by PF synapses and inhibition mediated by cerebellar cortical interneurons^33,46^. PCs send inhibitory projections to the deep cerebellar nuclei, and constitute the sole output of all motor coordination in the cerebellar cortex^33^. Here we performed a circuit-level investigation to link the PRRT2 deficiency-caused synaptic phenotypes with mouse dyskinesia phenotypes. Our experiments showed that PF-PC synapses of *Prrt2*-mutant mice exhibited a higher level of synaptic facilitation than those of WT mice, with increasing amplitudes of evoked EPSCs triggered by later stimuli. Particularly, we found that optogenetic stimulation of GCs could induce a much higher firing frequency of PCs in *GluN2C-iCre;Ai32*^+/−^;*Prrt2^−/−^* mice (Figure 6), compared with the *GluN2C-iCre;Ai32*^+/−^;*Prrt2*^+/+^ controls. Thus, our work provides a specific circuit-level mechanism underlying PRRT2-related behavioral phenotypes in which specific ablation of pre-synaptic PRRT2 in GCs facilitated transmission at PF-PC synapses, leading to enhanced PC firing and attacks of dyskinesia in the mutant mice.

We believe that our findings make many significant contributions to current understanding of the molecular, cellular and circuit mechanisms underlying PRRT2-related dyskinesia. As neuronal circuits regulating motor function vary largely between rodents and humans^31^, it would be of value to scrutinize more anatomical substrates that underlie the heterogeneous phenotypes of PRRT2-related disorders. Further research using non-human primates would offer an excellent way of clarifying these issues. In addition, the mechanisms by which PRRT2 regulates SNARE complex formation and synaptic transmission awaits further investigations.

## Materials and Methods

### Animals

*Prrt2^Stop^* knock-in mutant mice in a C57BL/6J background were generated by CRISPR/Cas-mediated genome editing, according to the previously described protocols^47^. Briefly, the Cas9 (Addgene #44758), sgRNAs (guide RNA target site: 5′-CGAGTTTCTGCAGCACACGGGGG-3′) and donor RNA (5′-CTCACCACCCTCAACTAAAACACCCCCAGCCAATGGGGCTCCCCCCTAGTAACGTGTGCTGCAGAAACTCGTTGAGGAAGACAGAATAGGAAGGGCAC-3′) were transcribed *in vitro* by T7 RNA polymerase as described before, and then purified using a MEGAclearTM Kit (Life Technologies, USA). Subsequently, mixtures (20 ng/μl for each) of Cas9 mRNA, sgRNAs and donor RNA were microinjected into the cytoplasm and the larger (male) pronucleus of 170 fertilized one-cell eggs from mice with a C57BL/6J background; the eggs were then transferred into the oviducts of pseudopregnant C57BL/6J female mice. After site-specific genome modification analysis by PCR and sequencing, of 34 viable pups born, 9 pups were identified to carry the correct mutant allele. Four F0 mice were selected for crossing with WT mice with the same C57BL/6J background to produce the F1 generation.

*LoxP*-flanked (floxed) *Prrt2* mice with a 129/C57 background were generated through homologous recombination with isogenic DNA constructs and then used to generate *Prrt2* cKO mice with Cre/*loxP* technology. Floxed mice were initially generated by Shanghai Biomodel Organism Science & Technology Development (SBOSTD) Co., Ltd. In brief, for the targeting vector, we cloned a 9-kb genomic fragment containing exon 2 of murine *Prrt2*, with a 2.8-kb genomic fragment upstream and a 3.1-kb downstream from exon 2, respectively, to act as the 5′ and 3′ homology arms. Exon 2 encoding the core region of murine *Prrt2* was flanked with a pair of *loxP* sequences (*Prrt2^loxP/loxP^*), and a neomycin resistance cassette (*Neo*) flanked with a pair of *FRT* sites was used for positive selection. The *Neo* cassette was ultimately removed by crossing with transgenic mice (SBOSTD Co., Ltd., China) carrying Flp recombinase prior to crossing with Cre transgenic mice. The *loxP* sites do not interfere with the normal expression of the target gene, but constitute a binding domain for the DNA recombinase Cre. Either transgenic mice carrying *Cre* allele under the control of a *Nestin* promoter (*Nestin-Cre*)^48^, CaMKIIα promoter (*CaMKIIα-iCre*)^49^ or a GluN2C promoter (*GluN2C-iCre* mice)^35^ were crossed with *Prrt2^loxP/loxP^* mice, thus selectively deleting exon2 of *Prrt2* in a subset of cells of homozygous cKO mice. As each of the *Cre* mice and floxed mice carrying two *Prrt2* alleles exhibited comparable expression levels of PRRT2 protein to WT (Figure 4F), the data from these three genotypes were pooled and used as littermate controls for cKO mice. The *Prrt2^loxP/loxP^* mice were stereotaxically injected with AAV-Cre expressing GFP into their cerebellum to induce the localized knockout of *Prrt2* gene.

Mice with *Prrt2^Stop^* mutations or conditional knockout of *Prrt2* gene were viable and fertile, exhibited no visible abnormalities, morbidity or premature mortality, and had normal life spans. The homozygous mutants and WT controls were obtained from heterozygous crossings and were born with the expected Mendelian frequency. The reproductive capacity of mutant mice (7 ± 2 normal-sized pups per litter for both *Prrt2^Stop^*; 8 ± 1 pups per litter for *Nestin-Cre;Prrt2^−/−^*) had no significant difference from that of the wild-type mice (9 ± 2 pups per litter).

We generated a mouse line *GluN2C-iCre;Ai32*^+/−^;*Prrt2^−/−^* in which ChR2 was specifically expressed and PRRT2 was specifically ablated in GCs, by crossing Ai32 transgenic mice^36^ with *GluN2C-iCre;Prrt2^−/−^* mice. *GluN2C-iCre;Ai32*^+/−^;*Prrt2*^+/+^ mice normally harboring allelic murine *Prrt2* gene were used as the control.

All the mutant and transgenic mice were identified by PCR amplification and DNA sequencing. All animal experiments and data analysis were performed by well-trained researchers blind to the mouse genotypes or treatments. For electrophysiological recording, EM observation, biochemical experiments and all behavioral tests, mice used for analysis were littermates derived from heterozygous crossings. Mice were housed in 12-h light/12-h dark cycle and had free access to food and water. For each line of mutant mice, both males and females were used equally for analysis. In the present study, the genotype of each animal was assessed twice using PCR and by sequencing genomic DNA isolated from tail tissue both before and after each experiment. The age of the mice used in this study is indicated in each section of the online methods. All efforts were made to minimize animal suffering. All animal experiments were approved by the Animal Care and Use Committee of the Shanghai Institutes for Biological Sciences.

### Preparation of PRRT2 antibodies

Rabbit polyclonal antibodies against distinct target polypeptide sequences in the N-terminal part or full length of murine PRRT2 were generated with Freund's adjuvant method. Briefly, the cDNA encoding the target polypeptide sequences of mouse PRRT2 were obtained by RT-PCR, and inserted into the pGEX-KG vector. Inducible expression and purification of recombinant proteins were carried out using the GST gene fusion system. For preparation of rabbit antibodies against PRRT2, New Zealand rabbits were injected with 0.5 mg recombinant protein with Freund's complete adjuvant. Three weeks later, 0.25 mg protein with Freund's incomplete adjuvant was injected, and this was performed again 2 weeks later. The specificity and efficiency of the produced antibodies were tested by western blot and immunohistochemistry (IHC) of WT and *Prrt2*-mutant mouse tissues. Specific antibodies detected a single band in immunoblots of WT brain extracts and cultured neurons transfected with the PRRT2-pCAG vector.

### Immunostaining

For light-microscopic IHC, brain sections (30 μm) were incubated in 0.01 M PBS supplemented with 3% hydrogen peroxide for 10 min to block endogenous peroxidase and then in a blocking buffer containing 5% BSA/10% normal goat serum/0.25% Triton X-100 for 60 min at room temperature to prevent nonspecific staining. Following this, IHC was conducted on these free-floating sections and staining was visualized with a standard ABC Elite Kit (Vector Labs).

For confocal microscopic double-labeling immunoﬂuorescence, brain sections were incubated in the blocking buffer for 60 min at room temperature and then in a solution containing primary antibodies (Supplementary information, Table S2) from different species simultaneously for two nights at 4 °C. After washing, sections were incubated with appropriate secondary antibodies from the Alexa Fluor series (Invitrogen, USA) for 45 min at 37 °C and then counterstained with Hoechst 33342 (1:5 000; #C10022, Beyotime) for 15 min at room temperature to identify cellular nuclei. After mounting the sections, we observed them under a fluorescent confocal microscope (A1R, Nikon).

### Immunoblotting

Mouse brain tissues and cultured cells were homogenized and lysed in RIPA buffer (20 mM Tris-HCl (pH 7.5), 150 mM NaCl, 1 mM EGTA, 1 mM EDTA, 1% Triton X-100, 1% sodium deoxycholate, 1 mM PMSF, 10 mg/ml aprotinin, 1 mg/ml pepstatin A and 1 mg/ml leupeptin). The equivalent denatured proteins were then resolved by SDS-PAGE, transferred and immunoblotted with primary antibodies, followed by secondary IgG antibodies conjugated with horseradish peroxidase. Finally, the blots were visualized by enhanced chemiluminescence. Immunoblotting with mouse anti-GAPDH or anti-β-actin was used to indicate protein loading. To study SNARE complex formation, the protein concentration of each lysates was determined, and the samples from each genotype were then divided in two tubes: one tube was boiled for 20 min, and the other was kept at room temperature.

### RNA extraction, cDNA synthesis, reverse transcription PCR and real-time PCR

Forebrain or cerebellum tissues were rapidly dissected on ice and homogenized in Trizol Reagent (Invitrogen, USA) at 4 °C. RNA was then extracted and dissolved in nuclease-free water to a final concentration of 1 mg/ml. Total RNA (2 μg) was reverse transcribed using the MLV-RT kit from Invitrogen and 1/20 of the products were used for PCR amplification. We performed real-time PCR analysis of *Prrt2* mRNA expression using a SYBR Green I Premix Ex Taq Kit (Takara, Shiga, Japan) and an ABI Prism 7000 thermocycler (Applied Biosystems), according to the recommendations of the manufacturer. Dissociation curve analysis was carried out after PCR amplification to confirm the absence of nonspecific amplification products and primer dimers. The results of real-time PCR were normalized to control values. Information of the primers used is provided in Supplementary information, Table S1.

### Immunolabeling EM

Mouse brains were prepared for immunolabeling EM. Briefly, 4-week-old mice were transcardially perfused with 4% paraformaldehyde (PFA) and 0.1% glutaraldehyde in 0.1 M PB for 30 min. Brains were removed after perfusion and post-fixed for 12 h at 4 °C. Coronal sections (100 μm thick) were sectioned using a vibratome (Leica VT1000 S).

For post-embedding immunogold labeling, brain sections crossing the cerebellum or cerebral cortex were post-fixed in 0.5% osmium tetroxide and processed for Epon 812 embedding. The ultrathin sections were stained with specific rabbit anti-PRRT2 antibodies (1:200; #3) and then goat anti-Rabbit IgG with 15 nm immunogold (1:100; #25113, EMS, USA).

For pre-embedding immunoperoxidase staining, vibratome sections of mouse cerebellum and cerebral cortex segments were immunostained with our rabbit anti-PRRT2 antibodies (1:200) followed by biotinylated goat anti-rabbit IgG (1:200) and then avidin-biotin-peroxidase complex (1:100; Sigma). The sections were incubated in 0.01 M PBS containing 3, 3′-diaminobenzidine and hydrogen peroxide. They were then post-fixed with 2.5% glutaraldehyde and 1% osmium tetroxide, dehydrated and processed for Epon 812 embedding. To distinguish the vesicles with PRRT2-immunoreactivity from unlabeled vesicles, we did not counterstain the ultrathin sections with uranyl acetate and lead citrate.

### Transmission EM

Four-week-old mice were used for transmission EM analyses. Mice were anesthetized with 5% urethane and perfused with saline (pH 7.4), followed by 2% PFA and 2% glutaraldehyde in 0.1 M PB (pH 7.4). Brains were removed, and 100 μm horizontal vibratome sections cut in 0.1 M PBS. The motor cortex and cerebellum lobes were dissected from desired sections, post-fixed for 1 h with 1% osmiumtetroxide in 0.1 M cacodylate buffer (pH 7.4), dehydrated in graded ethanol concentrations, rinsed in propylene oxide, and then embedded in Epon 812. Ultra-thin sections (60-80 nm) were cut and stained with uranyl acetate and lead citrate. Complete profiles of nonperforated asymmetric synapses on dendritic spines in the molecular layer of cerebellum were selected and photographed using a digital camera attached to a Hitachi 7500 electron microscope operated at 80 kV at a final magnification of 5 000-60 000. A total area of 6 460 μm^2^ from WT and 5 302 μm^2^ from *Prrt2^Stop^* mutant mice were counted for quantification of synapse density. A total of 202 asymmetric synapses from four WT mice, and 439 synapses from four mutants were analyzed. The small synaptic vesicles were defined into two groups to count as “docked” vesicles (located within 50 nm of the presynaptic active zone) and “reserved” vesicles (located 50-550 nm from the presynaptic active zone) according to criteria developed by Dickinson-Nelson and Reese (1983). We also normalized vesicle counts to the length of the active zone as determined by the length of postsynaptic density^24^. The investigator was blinded to the mouse genotypes.

### Three-dimensional reconstruction of serial EM images

3D rendering of synapses was reconstructed from the serial EM images of more than 500 sheets with 5 nm thickness obtained by 3VIEW focused ion beam/scanning EM (FIB/SEM) system (FEI, USA) in the FEI China-Shanghai facility, according to the manufacturer's protocol, as previously described^50^. In brief, the block face was trimmed using a sharp knife. SEM images of the untrimmed block face were used to select the desired field of view before the final trimming step, producing the desired small cut pyramid. About 800 serial images were taken using an environmental SEM Quanta FEG 200 (FEI, Netherlands) with a 3VIEW serial block face sectioning and imaging system (Gatan, USA) at an accelerating voltage of 5 keV. The equipped diamond knife used was custom made by diatome (Diatome, Switzerland), and the section thickness was 5 nm. Images were collected at an imaging size of 1 k × 1 k, pixel size of 2.47 nm, pixel dwell time of 80 μs, chamber pressure of 0.50 Torr and spot size of 3. Finally, serial images were aligned and 3D structures of more than three typical synapses in each group were rendered using an incorporated TrakEM2 plugin in combination with Imiris 8.0 software (Bitplane, Switzerland).

### Isolation of vesicles and immunoblotting

Synaptosomes were prepared from the forebrain and cerebellum of P28-P60 male mice. Mice were decapitated, and the brain tissue was rapidly removed and homogenized in preparation buffer (320 mM sucrose, 4 mM HEPES, pH 7.4) using a Teflon-glass homogenizer at 4 °C. The homogenate was centrifuged for 10 min at 1 000× *g*. The pellet was discarded and the supernatant was collected and centrifuged for 15 min at 9 200× *g*. The pellet was re-suspended and re-centrifuged for 15 min at 10 500× *g*. The resulting pellet was re-suspended in lysis buffer (5 mM Tris/HCl, pH 7.4) with an additional 9 volumes of ice-cold H_2_O to osmotically lyse the synaptosomes (20 up-and-down movements with a glass pipette) for 30 min. The lysate was then centrifuged at 100 000× *g* for 2 h and the pellet was re-suspended in sucrose buffer (200 mM sucrose, 0.1 mM MgCl_2_, 0.5 mM EGTA, 10 mM HEPES, pH 7.4). The final suspension was layered onto a continuous sucrose gradient ranging from 0.3 to 2.0 M sucrose/4 mM HEPES (pH 7.4), and the gradient was centrifuged at 100 000× *g* for 3 h. The protease inhibitors aprotinin, leupeptin, pepstatin and PMSF were added to all solutions. Samples were processed for SDS-PAGE, transferred, probed with antibodies, and visualized with enhanced chemiluminescence as described below.

### Nissl staining

Nissl staining was conducted on brain sections (30 μm) and transverse spinal cord sections as described previously^29^. Images were captured under a Nikon E600FN upright microscope using Neurolucida software (MBF science).

### Cell culture and transfection

Neurons were cultured from cortices of P0 mouse pups. Mouse glial cultures were prepared from cortices of P1-P2 pups. Neurons were electroporated with 2 μg plasmids using the Amaxa Nucleofector II System (Lonza Amaxa, Germany), according to the manufacturer's protocol, and cultured in neurobasal medium supplemented with B27 serum. Because the groups in experiments using cultured cells were defined by treatments, no randomization was used.

### Mass spectrometry and analysis

Protein complexes immunoprecipitated from mouse cortex tissue using our PRRT2 antibody were extensively washed with PBS, followed by an on-bead digestion protocol as described^23^. To summarize, beads were suspended in 8 M urea containing 10 mM DTT, and incubated at 37 °C for 30 min. The solution was then cooled to room temperature and iodoacetamide was added to a final concentration of 15 mM, and incubated at room temperature for 20 min in the dark. This solution was diluted to a final urea concentration of 2 M with 100 mM Tris-HCl. Proteins were subsequently digested with 1 μg of trypsin at 37 °C overnight. Digestion was terminated by adding formic acid (FA) to 5% and then centrifuged. Half of the peptide supernatant was used for liquid chromatography coupled with MS analysis to identify the proteins. Peptides were then resuspended in 2% acetonitrile and 1% FA, and loaded onto a microcapillary column packed in house with 15 cm of reversed-phase MagicC18 material (5 μm, 200 Å, Michrom Bioresources, Inc., Auburn, CA) using a built-in autosampler in a Proxeon NanoEasy LC 1000 HPLC system (Thermo Scientific, Bremen, Germany). The effective HPLC separation was performed with a 10%-35% MeCN (1% FA) gradient over 80 min followed by a 35%-98% MeCN (1% FA) over 20 min, after a 25 min isocratic loading at 2% MeCN and 1% FA. Mass spectra were acquired on a Q-Exactive mass spectrometer (Thermo Scientific, Bremen, Germany), with each duty cycle containing a MS scan at 70 000 resolution (at 200 *m/z*) followed by 10 MS/MS scans at 15 000 resolution (at 200 *m/z*).

The raw MS data were searched against the mouse IPI databases (version 3.86, released on 28 June 2012) using the MaxQuant software suite^51^ utilizing its LFQ feature. Cysteine residues were required to have a static modification of 57.021 Da and a differential oxidation (+15.995 Da) on methionine residues was permitted. The protein level false discovery rate was set to be < 1%. For LFQ, at least one peptide was required for the quantification, and iBAQ value calculation was selected.

Because 4 WT and 4 mutant brains were used for immunoprecipitation (IP) experiment followed by MS analysis, a Student's *t*-test was performed on each protein using the iBAQ values. Multiple hypothesis testing was performed following the initial test using the Benjamini-Hochberg (BH) procedure. To select high-confidence interacting partners with PRRT2, a iBAQ ratio between WT and mutant of ≥ 4 was set as the determining criteria, together with an adjusted *P*-value of < 0.05. To reduce the false-negative rate, we considered a medium confidence interacting partner with a WT and mutant iBAQ ratio of > 2 and the adjusted *P*-value between 0.05 and 0.1. A few proteins with *P*-values between 0.1 and 0.2 but only identified in WT samples were also included. We performed two additional sets of IP-MS experiments comparing protein complexes between WT and mutant cerebellum, using total spectral count as a quantitative measure. The shared proteins and their spectral count values were combined.

Both medium-confident and high-confident PRRT2 interacting partners were loaded into the String (string-db.org) protein-protein interaction database to build a binary protein-protein interaction network. The network was visualized using the VisANT platform.

### Co-IP

For IP experiments, brain lysates were homogenized in ELB buffer (100 mM Tris, 10 mM EDTA, 300 mM NaCl, 0.2% NP-40, pH 7.5), immunoprecipitated with anti-PRRT2 antibodies overnight at 4 °C and subsequently incubated with Protein G-Agarose (20 ml, Pierce) for 2 h at 4 °C. Following this, immunoprecipitates were collected and aspirated. The sepharose was then re-suspended in ELB buffer, washed at least 4 times, and incubated in SDS buffer for 5 min at 100 °C, and the supernatant was subsequently subjected to immunoblotting.

### Stereotaxic injection of engineered AAV, *in vivo* optogenetic manipulation

Engineered AAV expressing GFP (AAV-GFP) or a fusion construct of Cre recombinase and GFP (AAV-Cre-GFP), with a CMV promoter, was intra-cranially microinjected to infect neurons *in vivo* as previously described^52^. The use of GFP allowed the visualization of the infected neurons. Previous work had demonstrated that GFP expression did not interfere with the activity of Cre recombinase^52^.

For local knockout of *Prrt2* in cerebellum by viral injection, *Prrt2^loxP^*^/^*^loxP^* mice were either injected with AAV-CMV-GFP (control) or AAV-CMV-Cre-IRES-GFP (cKO). In brief, 6-week-old *Prrt2^loxP^*^/^*^loxP^* mice were deeply anesthetized with sodium pentobarbital (40 mg/kg body weight) by intraperitoneal injection and then placed on a stereotaxic apparatus. About 1 μl of purified and concentrated AAV (∼10^12^ infections units per ml) was injected stereotactically into the cerebellum vermis of mice at two sites (coordinates from bregma: 6.0 mm posterior, 0.0 mm lateral, 2.0 mm and 3.0 mm ventral), using glass microelectrodes at a slow rate (∼100 nl/min) under focal pressure controlled by a Picospritzer III (Parker, USA). The injection microelectrode was slowly withdrawn 5 min to ensure diffusion of the virus. After a 3-week recovery period, at least 3 mice were sacrificed for immunohistological analysis to verify the efficiency of virus transfection and Cre expression, and others were used for subsequent behavioral analysis. The injection sites were examined at the end of all the behavior tests.

For behavioral analysis of mutant mice with optogenetic manipulation, 1 μl of AAV expressing ChR2 (pAAV-hSyn-hChR2(H134R)-mCherry) (1.23 × 10^13^ infections units per ml) were stereotactically delivered into the cerebellar vermis of 6-week-old mice to infect cerebellar neurons (coordinates from bregma: 6.0 mm posterior, 0.0 mm lateral, 2.4 mm ventral). Optical fibers (Doric Lenses) were implanted 0.2 mm above the virus injection sites. Mice were then allowed to recover for 3 weeks. The specificity and efficacy of expression of opsins in neurons have been previously characterized^52^. The *in vivo* light stimulation procedure was then administered. To stimulate cerebellar neurons, blue light flashes (473 nm wavelength; 5 mW) lasting 10 ms at 40 Hz programmed by Master 8 through DPSSL laser system (Laser Century) were delivered *in vivo* for 10 s per trial. No more than 10 trials with a 10-s interval were delivered into each mouse. All homozygous mutants we tested were induced to dyskinesia with three trials of optical stimulations, however, even 10 trials of optical stimulations failed to induce visible behavioral abnormalities in WT mice. Histological analysis showed that the treatment did not lead to defects in the structural integrity or substantial cell death of GCs in the cerebellum. All quantifications were performed by investigators blind to the experimental conditions.

### Electrophysiology analysis

Animals (P16-21) were housed under standard conditions and were processed for acute slice preparation as previously described^53^. The investigators were blinded to the mouse genotype when performing electrophysiological recording and analysis. In brief, mice were decapitated and their brains quickly were removed into ice-cold sucrose-based ACSF containing 2.5 mM KCl, 1.25 mM NaH_2_PO_4_, 26 mM NaHCO_3_, 3 mM MgSO_4_, 0.1 mM CaCl_2_, 10 mM dextrose, 213 mM sucrose, 2 mM sodium pyruvate, 0.4 mM sodium ℒ-ascorbate, and 0.05 mM D-APV (Tocris).

Electrophysiology experiments were performed on transverse slices of cerebellar vermis (lobules 4-6)^34^. The slices (300 μm) were cut using a vibrotome (Leica VT1000S, Germany) and quickly transferred to a chamber containing oxygenated ACSF containing 126 mM NaCl, 2.5 mM KCl, 1.25 mM NaH_2_PO_4_, 1 mM MgSO_4_, 2 mM CaCl_2_, 26 mM NaHCO_3_, 0.01 mM sodium pyruvate and 25 mM dextrose (pH 7.4, 320 mOsm) for 30 min at 34 °C. Subsequently, slices were kept and all recordings were carried out at room temperature (20-25 °C). The recording chamber was continuously perfused at a rate of 2 ml/min with the oxygenated ACSF, bubbled with 95% O_2_ and 5% CO_2_. Unless otherwise stated, picrotoxin (100 μM) was added to the bath solution to block fast inhibitory transmission.

Patch pipettes were prepared from borosilicate glass capillaries and had 2.0-4.0 MΩ resistances with an internal solution containing 115 mM CsMethanesulfonate, 20 mM CsCl, 10 mM HEPES, 10 mM EGTA, 2 mM MgSO_4_, 5 mM ATP (magnesium salt), 0.4 mM GTP (sodium salt), 10 mM phosphocreatine, 20 mM tetraethylammonium-Cl and 4 mM QX-314Cl (pH 7.4, 300 mOsm). PCs were voltage-clamped at −70 mV in the whole-cell configuration with a MultiClamp 700B amplifier (Axon Instruments, Union City, CA, USA). Signals were filtered at 4 kHz and digitized at 10 kHz with a DigiData 1440A (Axon Instruments, USA). Data were acquired and analyzed with pClamp10.3 software (Axon Instruments, USA).

To evoke PF-EPSC, a stimulus glass electrode (tip diameter, 5-10 μm) filled with ACSF was positioned on the molecular layer surface to stimulate PFs at ∼100 μm away from the recorded PC. The stimulus intensity (< 50 μA, 0.1 ms) was set at the beginning to produce a PF-EPSC between 100 and 200 pA (< 30% of the maximum response) in all the experiments. Stimulation frequency was 0.1 Hz or is mentioned in the text if otherwise. Recordings were made in lobules 3-8 of the vermis cortex. Series resistance was held between 5 and 10 MΩ, and monitored every 10 min. If the resistance changed more than 20%, the record was discarded.

Spontaneous and miniature EPSCs were analyzed with IGOR Pro (Wavemetrics). Events were detected using SpAcAn, a custom-made threshold detection algorithm^54^. Additional chemicals were used at the following final concentrations in the bath as indicated: 50 μM D-APV, 5 μM NBQX and 1 μM TTX. The above chemicals were obtained from Sigma or Tocris.

For PC spontaneous firing recordings, the recording electrodes were filled with an internal solution (pH 7.3, 295-310 mOsm) containing 130 mM K-gluconate, 10 mM KCl, 10 mM Hepes, 0.5 mM EGTA, 2 mM MgSO4, 4 mM ATP.Na2, 0.3 mM GTP and 10 mM Phosphocreatine. Optical stimulation was delivered from a microscope-mounted blue LED (X-Cite, Lumen Dynamics, Canada) through the objective lens directed onto the slice. The power of the LED light was < 3 mW. PCs were cell-attached patched in voltage-clamped mode, and held at 0 mV.

### Kindling procedure

The kindling model was conducted according to our protocol previously described^29^. Briefly, a bipolar electrode was stereotactically implanted into the right amygdala of adult animals under sodium pentobarbital (40 mg/kg, i.p.; Sigma) anesthesia, for stimulating and recording, using the following coordinates (with bregma as the reference): 2.8 mm posterior, 4.9 mm lateral and 8.6 mm below dura for rats; 1.2 mm posterior, 2.8 mm lateral and 4.9 mm below dura for mice. Four screws were inserted into the skull through a drilled hole without piercing the dura, with one of them on the left (coordinates from bregma: 4.0 mm posterior and 2.0 mm lateral) serving as the ground electrode. After a post-surgery recovery period of 1 week, the electrographic seizure threshold (EST) was determined and stimulations at EST current intensity were subsequently administered following the standard amygdala kindling protocol. EEG and behavioral seizures were observed and recorded. Behavioral intensities were scored according to Racine's standard classification^29^. No behavioral or EEG abnormalities were found among different genotypes before electrical stimulation using the kindling paradigm.

### Behavioral monitoring and classification

To observe PKD-like behavior in mutant mice, mice received monitoring continuously with closed circuit television to record the occurrence of dyskinesia. To observe expected spontaneous dyskinesia in mutant mice, both male (eight for *Prrt2^Stop^*, four for *Nestin-Cre;Prrt2^−/−^*) and female mice (eight for *Prrt2^Stop^*, four for *Nestin-Cre;Prrt2^−/−^*) at 3 weeks of age received monitoring continuously with closed circuit television for 2 months (8 h/day, 5 days/week) to record the occurrence of dyskinesia. The severity of induced dyskinesia in mice was evaluated using a modification of a previously published scale^55^ as follows: 0, normal motor behavior; 1, slightly slowed or abnormal gait; 2, mild motor impairment with transient abnormal postures, infrequent tremors and/or infrequent falls; 3, moderate motor impairment with falling down, loss of postural control and/or frequent tremors; and 4, severe motor impairment with rigor in limbs and tail, clonic limb shaking or sustained dystonic postures. Scoring was conducted by well-trained researchers blinded to the mouse genotype and treatment whenever possible.

### Drug delivery

PTZ is a non-competitive GABA antagonist which, at the appropriate dosage, induces intense temporary twitches and seizures lasting several minutes with a short latency of several minutes. For PTZ treatment, PTZ (Sigma, MO) dissolved in 0.9% sterile saline was administered intraperitoneally at dosage of 40 mg/kg body weight. Animals were then observed continuously for behavioral seizures and occurrence of dyskinesia using closed circuit television combined with EEG recording.

To test whether CBZ has an effect on the dyskinesia symptoms of *Prrt2*-mutant mice, CBZ was injected intraperitoneally at the specified time points at different doses of either 1, 5, 15, 30 mg/kg body weight, of which 15 mg/kg was found to have appropriate action with significant inhibitory effect on dyskinesia in mutant mice but no obvious effect in WT mice. For PTZ model, *Prrt2^Stop^* mice were administrated CBZ about 6 min later after the PTZ injection when they began to recover after a generalized seizure.

### Behavioral tests

Six-week-old mice of different genotypes were evaluated for each behavioral test including beam balance test, rotarod test, open field test, olfaction test, elevated plus maze, acoustic startle, tail suspension, and fear conditioning test. Observers were blinded to the mouse genotype during all testing. Behavioral tests were performed in the behavioral test core of the Institute of Neuroscience of Chinese Academy of Sciences and analyzed with automated system.

### Beam balance test

The beam apparatus consisted of 100-cm square beams of three different widths (28 mm, 12 mm and 5 mm), resting 50 cm above the ground, supported by two poles. A black box was placed at the end of the beam to act as the end goal. Mice were placed at start of the beam with a bright light positioned behind them. The time taken to cross the center 80 cm of the beam was scored. Mice were trained prior to the initial test for 2 days on all beams with 15-min inter-trial intervals. On testing days, three trials were performed on each mouse with the same resting interval time between beams. If a mouse took longer than 60 s to cross or fell off the beam, it was scored as 60 s. Trials in which they stopped or turned around were repeated. A thick blanket placed below was used to prevent injury.

### Rotarod test

This test was used to measure motor balance and coordination by placing mice on an accelerating, 3 cm diameter rotating rod (Ugo Basile, Italy) for three trials, with a minimum 15-min interval between each trial. The rotarod was started at 4 rpm and increased to 40 rpm over a period of 5 min. The mean latency to fall off during the three trials was recorded for analysis. Mice from each group were pre-trained for adaptation at the first day and recorded at the subsequent days.

### Open field test

This test was used to measure the exploratory locomotor activity. Each mouse was placed in the center of a transparent plastic chamber (40 × 40 × 40.5 cm) and allowed to explore freely for 15 min. The testing arena was brightly lit. During each session, their behavior was automatically videotaped and subsequently analyzed using the EthoVision video tracking system.

### Olfaction test

Olfaction test was assessed by measuring latency to find a buried piece of food in a cage to which mice had been previously habituated. The testing cage consisted of a plastic arena (45 × 45 × 15 cm), where floor was covered with an ∼2-cm depth of corncob bedding. Mice were deprived of food in their home cage for 24 h prior to testing. After buried food was placed on a circular piece of white filter paper (12 cm in diameter) positioned in the center of the arena, the mouse was placed in a corner of the arena. The latency to approach the food and begin feeding was recorded (for a maximum time of 10 min).

### Elevated plus maze

The elevated plus maze comprised of four perpendicular arms including two closed arms (30 × 6 × 20 cm), two open arms (30 × 6 cm) and a central platform (6 × 6 cm). The maze was placed at a height of 50 cm with normal light. Each animal was placed in the central platform of the maze and allowed to explore the apparatus freely for 5 min. The EthoVision video tracking system and software was used for recording and data analysis. The percent of time that each mouse spent in the open arms and the closed arms was measured.

### Acoustic startle and PPI

The startle reflex apparatus (ASR-PRO1, MED Associates) was used to measure the prepulse inhibition of startle (PPI) of mice. Mice were placed in the measurement box individually. For the acoustic startle, the response to a stimulus of loud noise (120 dB) was recorded. To determine the degree of PPI, the startle-eliciting stimulus (120 dB sound) was preceded by a brief low-intensity stimulus of either 78 dB, 81 dB or 84 dB. Then the new startle response at each intensity level was measured and analyzed. During the test, background sound level was set at 64 dB.

### Fear conditioning test

The Video Tracking of Fear Conditioning System (VFC-008-LP, MED Associates) was used to measure conditioned immobility or “freezing”. In training procedure, each mouse was placed into the shock box and allowed to explore freely for 2 min. Subsequently, a white-noise tone (87 dB) sounded for 30 s (conditioned stimulus). During the last 1.5 s of the tone, mice received a mild foot shock (0.5 mA, unconditioned stimulus). A same tone-foot shock (conditioned-unconditioned stimulus) combination was delivered again 2 min later. One minute later, the mice were returned to their home cages. 22 h later, the trained mice were taken back for context test. During the test, mice were individually placed back into the same training box, and monitored by an overhead near-infrared camera in the box for 5 min. The cue test was performed 2 h after the context test and data were collected for analysis.

### Tail suspension

An automated tail suspension system (TAILSUSP-1N96, MED Associates) was used to measure the immobility of suspended mice. The animal's tail was wrapped by adhesive tape at a constant site three quarters of the distance from the base of the tail. The mice were then suspended by passing the suspension hook through a metal chain and the total time of immobility was recorded for analysis automatically.

### Statistical analyses

All experiments and data analysis were performed by investigators blind to the mouse genotypes and treatments. All computed parameters were quantified and compared between tested groups unless specified otherwise. No animals were excluded from the analysis. Data were presented as mean ± SEM, except for the percentage of animals, data of mass spectrum and stimulation numbers in the kindling model. The sample size of each experimental group was selected to minimize the number of mice used in the studies while providing sufficient information to report significant and reliable results. No statistical method was used to predetermine sample size. Significance between two means was analyzed using two-sided unpaired Student's *t*-test. Comparisons between multiple groups were assessed by one-way ANOVA with Dunnett's *post hoc* analysis or two-way ANOVA with Bonferroni *post hoc* test. The nonparametric Mann-Whitney *U*-test was used in comparisons of < 6 cases. We used Fisher's exact test to calculate *P*-values comparing two ratios. All statistical analyses were performed using SPSS 15.0 software. *P*< 0.05 was considered statistically significant.

## Author Contributions

Z-YW and Z-QX initiated the project. G-HT and Y-YL did major experiments and analyzed the data. LW and Y-YL carried out electrophysiological experiments; KL performed optogenetic manipulation and video processing; KS provided *GluN2C-iCre* mice. G-HT and Y-YL interpreted the data and wrote the manuscript. Z-QX supervised and organized the project. Other authors contributed to experiments and discussed the data.

## Competing Financial Interests

The authors declare no competing financial interests.

## Supplementary Information

Supplementary information, Figure S1 Generation of rabbit anti-PRRT2 antibody and characterization of PRRT2 protein.

Supplementary information, Figure S2 Expression pattern of endogenous PRRT2 in developing mouse brain.

Supplementary information, Figure S3 Generation of mutant mice to mimic the hot-spot mutation of human *PRRT2* in patients with PKD.

Supplementary information, Figure S4 PRRT2 protein was depleted in brains of *Prrt2*^stop^ mouse.

Supplementary information, Figure S5 PRRT2 inhibits SNARE complex formation both *in vivo* and *in vitro*.

Supplementary information, Figure S6 EM analysis of *Prrt2*^Stop^ synapses in mouse cerebellum.

Supplementary information, Figure S7 Behavioral profile of *Prrt2*-mutant mouse.

Supplementary information, Figure S8 Impaired performance of *Prrt2*-mutant mice in beam walking and Rotarod test.

Supplementary information, Figure S9 Generation and verification of *Nestin-Cre;Prrt2^−/−^* mice.

Supplementary information, Figure S10 Effect of CBZ on experimentally induced dyskinesia in mouse models.

Supplementary information, Figure S11 Increased short-term facilitation of PF-PC synapses in cerebellum of *GluN2C-iCre;Prrt2^−/−^* mice.

Supplementary information, Movie S1 3D Reconstruction of synaptic terminals in *Prrt2*^Stop^ and WT mouse cerebellum. Shown is 3D reconstruction of typical asymmetry excitatory synapses in the molecular layer of cerebellum from WT and *Prrt2*^Stop^ mutant mice, respectively, using serial EM images. Docked synaptic vehicles were labeled in green. More than three synapses were reconstructed in each block for analysis.

Supplementary information, Movie S2 Spontaneous paroxysmal dyskinesia in *Prrt2*-deficient mice. Shown are representative spontaneous dyskinesia attacks in distinct *Prrt2*-mutant mice occasionally observed in our experiments. The onset of the attack was signaled by contraction of the limbs, causing a rigid posture with lost mobility and no ambulation under nudge. As attacks progressed, the mouse lost balance, and began to slowly extend and contract its limbs more severely. Sometimes, mice exhibited stiffened postures and akinesia phenotypes. These episodes occurred paroxysmally and the attacks generally continued no more than 30 min. After that, the mouse recovered normally, with mobility restored comparable to the control littermates. These spontaneous attacks were never observed in WT mice.

Supplementary information, Movie S3 Dyskinesia attacks triggered by kindling-induced generalized seizures. The former part shows typical phenotypes of a WT mouse in the kindling model. Electric stimulation delivered into the mouse brain could immediately induced robust behavioral seizures lasting about 40 seconds, with typical epileptiform EEG discharges. When the seizures terminated, the mouse recovered to freely move normally. The later part shows paroxysmal dyskinesia attacks in a *Prrt2*^Stop^ mouse which was triggered rapidly after kindling stimulation-induced seizures. Like WT mouse, the mutant exhibited epileptic seizures after electric stimulation. However, dozens of seconds after strong behavioral seizures terminated, the mutant mouse began to exhibit dyskinesia-like behaviors. This time, the dyskinesia attacks lasted for about 2.5 h with a typical paroxysmal pattern, without epileptiform discharges in EEG recording.

Supplementary information, Movie S4
Dyskinesia attacks triggered by PTZ-induced generalized seizures. After intra-peritoneal administration of PTZ, class-5 generalized seizures were induced in both WT mouse (the former half part of the movie) and *Prrt2*^Stop^ mutant (the latter half part of the movie) in several minutes. The control WT mouse recovered quickly in a dozen of minutes after seizure behaviors. In contrast, dyskinesia occurred after PTZ-induced seizures in mutant mice, and lasted for more than 1 h.

Supplementary information, Movie S5 Dyskinesia attacks after thermal elevation. As shown in the movie, a homozygous *Prrt2*^Stop^ mutant mouse exhibited typical paroxysmal dyskinesia after 5-min exposure to 50 °C ambient temperature. As a control, a WT littermate beside it under the same condition exhibited normal motor behavior.

Supplementary information, Movie S6 EEG recordings for induced dyskinesia models. In the first section, the mutant mouse exhibited typical epileptic discharges and simultaneous epileptic seizures 2 min after the PTZ injection. Of note, there were no typical epileptiform discharges during the PTZ-induced dyskinesia attacks and inter-ictal period. In the second section, EEG was recorded in the forebrain of a *GluN2C-iCre;Ai32^+/−^;Prrt2^−/−^* mouse before and after the optogenetic stimulation, and no epilepsy features were observed in EEG recording. In the third section, a *GluN2C-iCre;Ai32^+/−^Prrt2^−/−^* mouse and a control mouse were placed in boxes with 50 °C ambient temperature for 5 min; thereafter, we recorded their EEGs and found no typical epileptic discharges during the occurrence of dyskinesia.

Supplementary information, Movie S7 Optogenetic stimulation-induced dyskinesia in *GluN2C-iCre;Prrt2^−/−^* mice. After three trials of 40 Hz blue light flashes with 10-second interval, the *GluN2C-iCre;Prrt2^−/−^* mouse showed paroxysmal dyskinesia that lasted more than 4 h. The onset of attacks immediately followed light stimulations. The same optogenetic stimulation conditions never induced dyskinesia in the WT mice.

Supplementary information, Table S1 Sequences of PCR Primers Used in This Study

Supplementary information, Table S2 Commercial Antibodies Used in Western Blot and IHC Experiments.
