# Supplementary material for: PRRT2 deficiency induces paroxysmal kinesigenic dyskinesia by regulating synaptic transmission in cerebellum
Source: Cell Res. 2017 Oct 20;28(1):90–110. doi: 10.1038/cr.2017.128 (PMC5752836; doi:10.1038/cr.2017.128)
Supplement: Supplementary information, Figure S1 — Generation of rabbit anti-PRRT2 antibody and characterization of PRRT2 protein. [file cr2017128x1.pdf]

Supplementary information, Figure S1

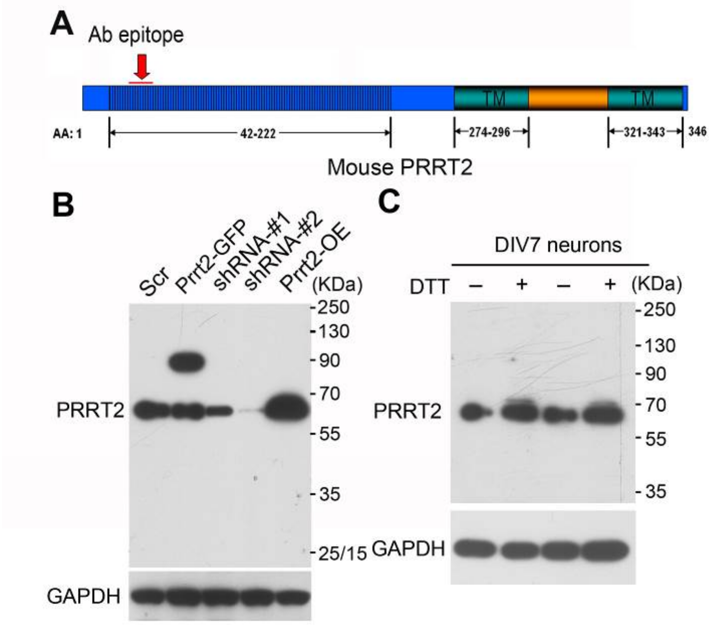

Figure S1 Generation of rabbit anti-PRRT2 antibody and characterization of PRRT2 protein. (**A**) Diagram of the targeted regions of our polyclonal rabbit anti-mouse PRRT2 antibody. (**B**) Verification of PRRT2 band in western blot using specific shRNAs, GFP-fused constructs, full-length constructs of mouse PRRT2 that were transfected into cultured mouse cortical neurons. GAPDH was used as a loading control. (**C**) Western blot analysis of dithiothreitol (DTT)-treated neuronal cultures using PRRT2 antibody. The immunoreactive band had no changes after treatment with DTT, excluding the possibility of di-sulfide bond mediated dimerization.
