# Supplementary material for: PRRT2 deficiency induces paroxysmal kinesigenic dyskinesia by regulating synaptic transmission in cerebellum
Source: Cell Res. 2017 Oct 20;28(1):90–110. doi: 10.1038/cr.2017.128 (PMC5752836; doi:10.1038/cr.2017.128)
Supplement: Supplementary information, Figure S2 — Expression pattern of endogenous PRRT2 in developing mouse brain. [file cr2017128x2.pdf]

Supplementary information, Figure S2

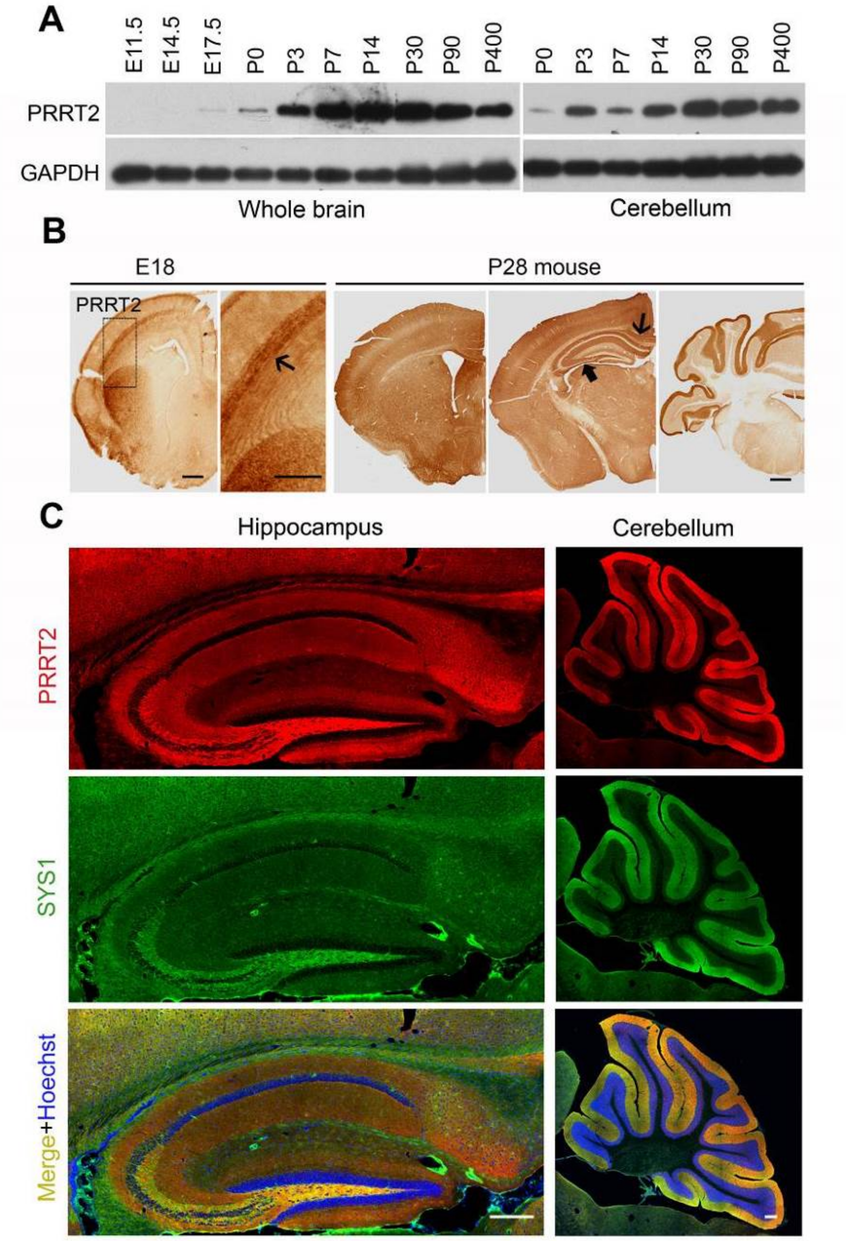

Figure S2 Expression pattern of endogenous PRRT2 in developing mouse brain. (**A**) Immunoblotting analysis of PRRT2 protein expression in brain lysates from mice at the indicated developmental stages. GAPDH served as an internal control. (**B**) IHC staining of PRRT2 on coronal mouse brain sections during development. Thin arrow points to corpus callosum with high immunoreactivity and thick arrow points to hippocampal mossy fibers. E18, embryonic day 18; P28, postnatal day 28. Scales bars,

200 μm for E18 and 500 μm for P28. (**C**) Co-staining of PRRT2 and synapsin1 (SYS1) in the P28 mouse brain sections. Hoechst33342 was used to identify the pyramidal cell layer (left panel) or granular cell layer (right panel). Note there were no PRRT2 signals but existed SYS1 signals in deep nucleus of cerebellum (right pannel). Scale bar, 200 μm.
