# Supplementary material for: PRRT2 deficiency induces paroxysmal kinesigenic dyskinesia by regulating synaptic transmission in cerebellum
Source: Cell Res. 2017 Oct 20;28(1):90–110. doi: 10.1038/cr.2017.128 (PMC5752836; doi:10.1038/cr.2017.128)
Supplement: Supplementary information, Figure S3 — Generation of mutant mice to mimic the hot-spot mutation of human PRRT2 in patients with PKD. [file cr2017128x3.pdf]

Supplementary information, Figure S3

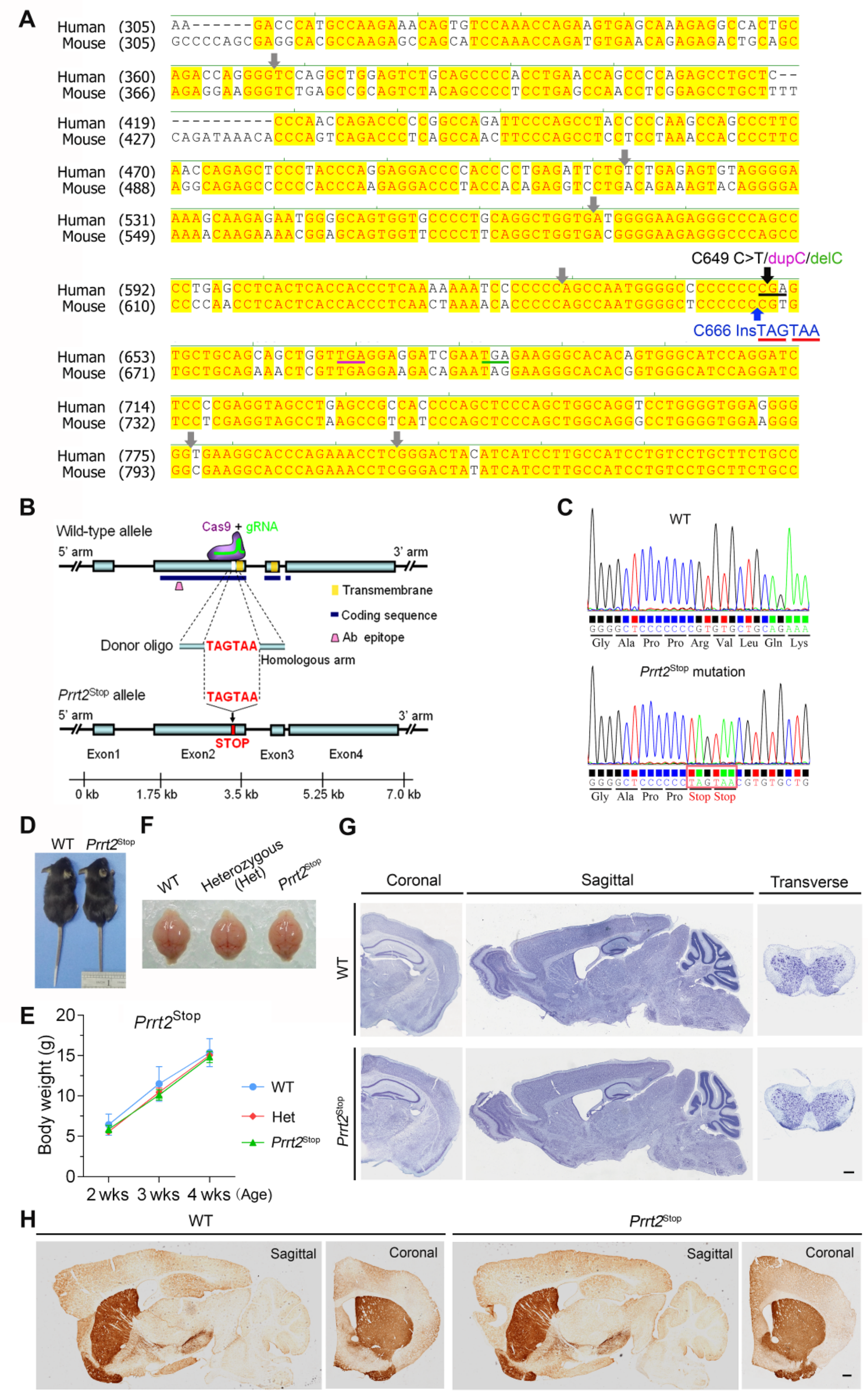

Figure S3 Generation of mutant mice to mimic the hot-spot mutation of human *PRRT2* in patients with PKD. (**A**) Shown is partial sequence alignment of human *PRRT2* (NM_145239.2) and mouse *Prrt2* (NM_001102563.1), indicating their identical overall organization and strong sequence conservation. Identical nucleotide sequences are shaded in yellow. A mutational hotspot (black arrow) of nine cytosines (c.649) in human *PRRT2* was responsible for most PKD cases, introducing a stop codon (underlined by black, pink, green bars, respectively) in advance prior to the sequences coding two transmembrane domains (807-867 and 954-1014, respectively). We thus planned to generate *Prrt2*-mutant mice with insertion of two stop codons (red bars) into C666 sites (blue arrow) of murine *Prrt2* to mimic the hot-spot nonsense mutation at the C649 site of human *PRRT2* gene in PKD patients. Some of other mutated sites found in PKD patients are indicated by gray arrows. (**B**) Schematic diagrams of generation of mutant mouse with incorporation of two stop codons (TAGTAA) into its endogenous *Prrt2* gene (referred to as *Prrt2*^{Stop}). Cas9/Crispa-mediated gene-targeting strategy was used to insert stop codons into murine *Prrt2*. Nucleotide sequence coding the epitope of specific PRRT2 antibody used in this study is indicated by pink trapezoid box. (**C**) DNA sequencing confirmed the correct incorporation of exogenous nucleotides into the target site of *Prrt2*^{Stop} mice. (**D**) No obvious changes in gross morphology of the mutant mice. (**E**) No significant changes in body weight (n=7, 14, 8 for WT, heterozygous and homozygous mice, respectively) of the mutant mice. Error bars, mean ± SEM. (**F**) Comparison of brains of mutant mice and their control littermates at 1 month of age. WT, wild-type; Het, heterozygous. (**G**) Nissl staining of sections from the mouse brains (left panel, coronal; middle panel, sagittal) and spinal cord (right panel) with the indicated genotypes showed that the gross anatomic structure of central nervous system was not apparently altered in *Prrt2*^{Stop} mutant mice. Scale bars: 600 μm, for brain; 300 μm, for spinal cord. (**H**) Tyrosine hydroxylase (TH) staining of dopaminergic neurons in the substantia nigra and terminals in the striatum showed comparable patterns in both WT and *Prrt2*^{Stop} mice. Scale bar, 400 μm.
