# Supplementary material for: PRRT2 deficiency induces paroxysmal kinesigenic dyskinesia by regulating synaptic transmission in cerebellum
Source: Cell Res. 2017 Oct 20;28(1):90–110. doi: 10.1038/cr.2017.128 (PMC5752836; doi:10.1038/cr.2017.128)
Supplement: Supplementary information, Figure S4 — PRRT2 protein was depleted in brains of Prrt2stop mouse. [file cr2017128x4.pdf]

Supplementary information, Figure S4

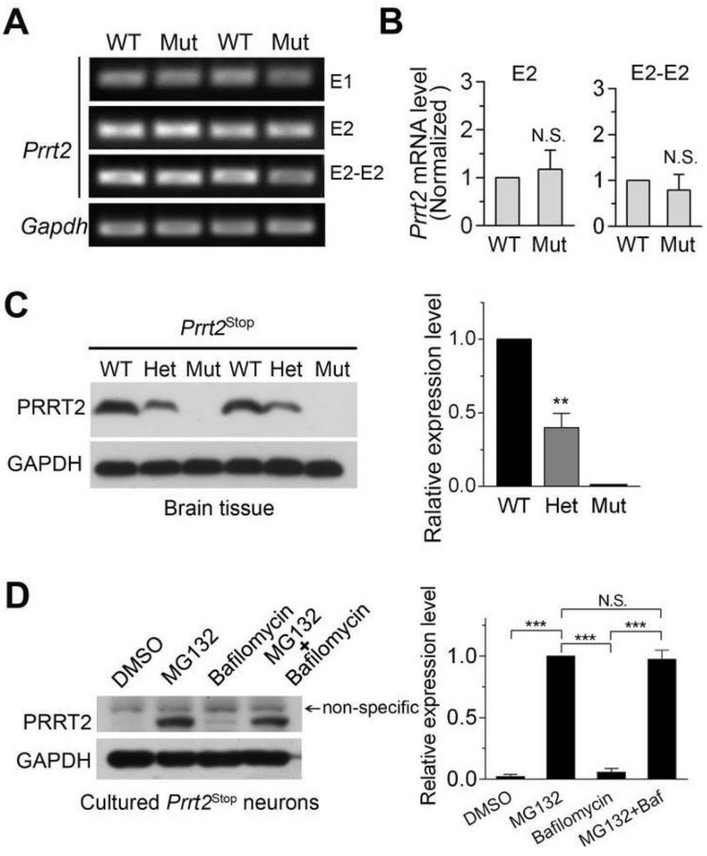

Figure S4 PRRT2 protein was depleted in brains of *Prrt2*^{stop} mouse. (**A**, **B**) Reverse transcription-PCR (**A**) and Real-time PCR (**B**) was used to detect the expression levels of *Prrt2* mRNA in mouse brains. PCR results revealed normal expression of *Prrt2* mRNA in *Prrt2*^{Stop} mouse brains. The exons that primers target are shown on the right. The targeted region of primers E2 spans the mutant sites; primers E2-E2 locate downstream of the mutant sites. Error bars, mean ± SEM; N.S., not significant. (**C**) Western blot analysis reveals a gene-dose dependent expression pattern of PRRT2 protein in brains. Brain lysates were from WT, heterozygous (het) and homozygous mutant mice, respectively. GAPDH was used as the loading control. Left panel, representative western blots; right panel, results of statistical analysis. $n = 4$ for each group. $^{**}P = 0.0084$; Students' *t*-test. (**D**) DIV7 neurons cultured from *Prrt2*^{Stop} mouse brains were treated with MG132 (25 μM) and Bafilomycin (50 nM), respectively. Application of MG132 could induce the specific expression of truncated PRRT2 in neuronal cultures from *Prrt2*^{Stop} mice, indicating proteasome-mediated

degradation of truncated PRRT2. Left panel, representative Western blots; right panel, statistical analysis of the expression levels for the PRRT2 residues. N.S., not significant. ***$P < 0.001$; Student's *t*-test.
