# Supplementary material for: PRRT2 deficiency induces paroxysmal kinesigenic dyskinesia by regulating synaptic transmission in cerebellum
Source: Cell Res. 2017 Oct 20;28(1):90–110. doi: 10.1038/cr.2017.128 (PMC5752836; doi:10.1038/cr.2017.128)
Supplement: Supplementary information, Figure S5 — PRRT2 inhibits SNARE complex formation both in vivo and in vitro. [file cr2017128x5.pdf]

Supplementary information, Figure S5

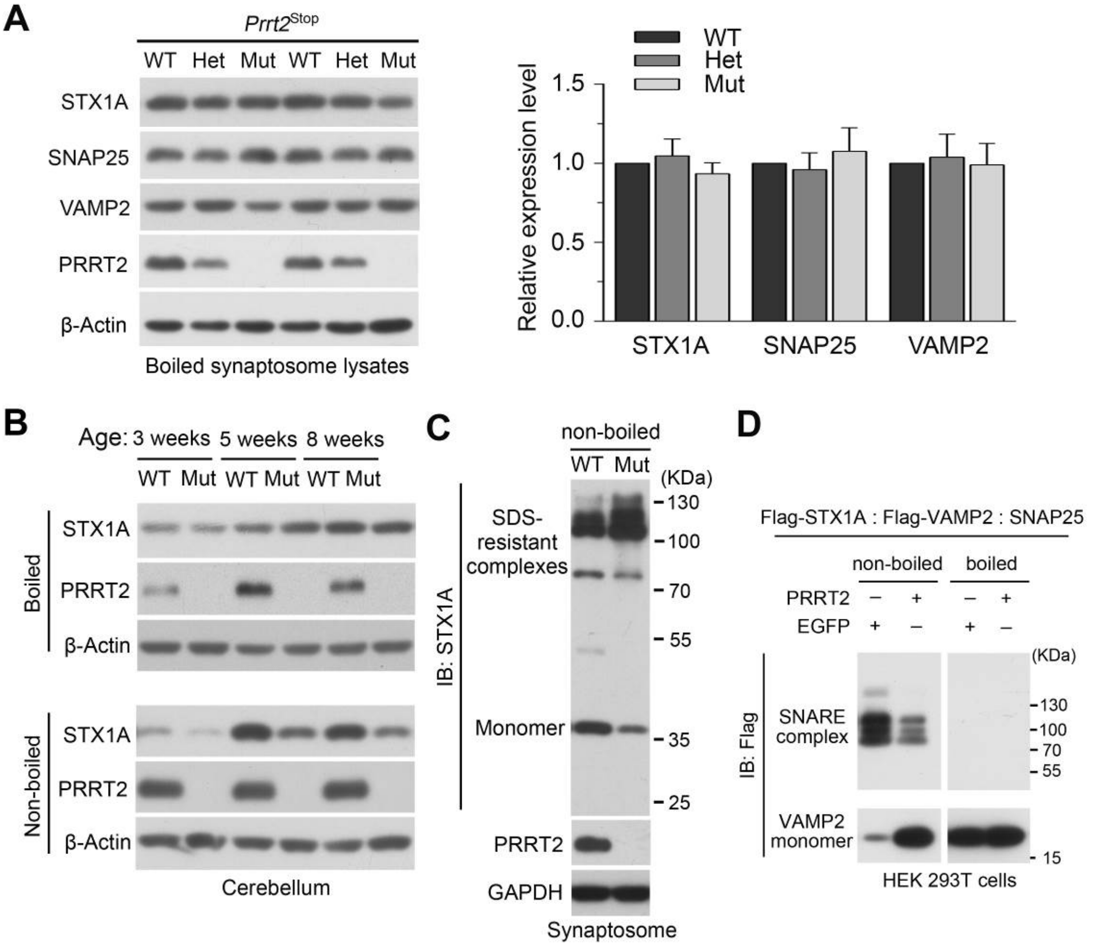

Figure S5 PRRT2 inhibits SNARE complex formation both *in vivo and in vitro*. (**A**) Representative western blots showing individual expression levels of the indicated synaptic proteins in boiled lysates from mutant and WT mouse brains. Immunoblotting analysis of boiled brain lysates revealed no significant difference in the protein levels of the SNARE complex components among the brains of different genotypes. (**B**) Immunoblots of monomeric STX1A using boiled or non-boiled cerebellar lysates from mutant (Mut) mice and their WT littermates at the indicated ages. Immunoblotting analysis of boiled brain lysates revealed no significant difference in the protein level of STX1A (upper panels); however, when using non-boiled brain lysates, we found surprisingly that the amount of monomeric STX1A markedly decreased in the mutant brain (lower panels). (**C**) Representative full-gel immunoblots of non-boiled synaptosomes from WT and *Prrt2*^{Stop} mutant mice showing the SDS-resistant SNARE

complexes identified by a STX1A antibody. Since assembled SNARE complexes are resistant to 1% SDS lysis buffer at room temperature but could be dissociated into monomeric SNARE proteins at 100 °C, we compared the amount of assembled SNARE complexes using synaptosome samples from WT and *Prrt2*^{Stop} mice both at room temperature. Results showed that the amount of assembled SNARE complex was significantly increased in the brains of mutant mice, accompanied simultaneously with a decrease in monomeric STX1A. (**D**) PRRT2 inhibits the formation of SNARE complexes in HEK293T cells transfected with all plasmids encoding exogenous SNARE complex components.
