# Supplementary material for: PRRT2 deficiency induces paroxysmal kinesigenic dyskinesia by regulating synaptic transmission in cerebellum
Source: Cell Res. 2017 Oct 20;28(1):90–110. doi: 10.1038/cr.2017.128 (PMC5752836; doi:10.1038/cr.2017.128)
Supplement: Supplementary information, Figure S6 — EM analysis of Prrt2Stop synapses in mouse cerebellum. [file cr2017128x6.pdf]

Supplementary information, Figure S6

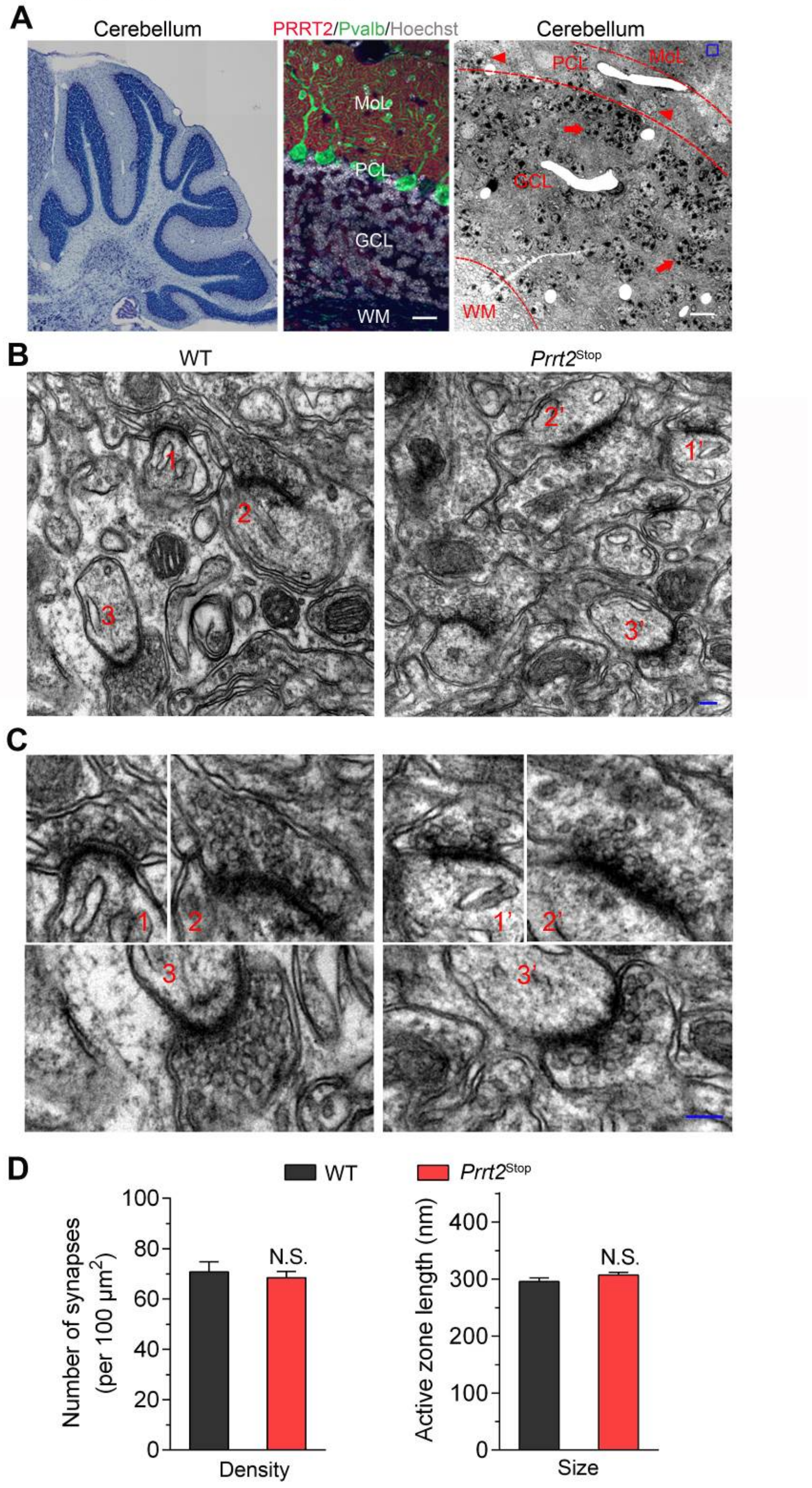

Figure S6 EM analysis of *Prrt2*$^{\mathbf{Stop}}$ synapses in mouse cerebellum. (**A**) A schematic diagram for capturing transmission electron microscopic (TEM) images of asymmetrical excitatory synapses in the molecular layer of mouse cerebellum in which PRRT2 is predominantly expressed. Arrows point to the nucleus of granular cells,

while arrowheads point to that of purkinje neurons. Blue box in the molecular layer (Mol) indicates the approximate position of observed area in each cerebellar slice under higher magnification. Scale bars, 30 μm (left) and 6 μm (right). (**B**) Representative TEM pictures of high magnification from the cerebellar molecular layer of *Prrt2*-mutant and WT mice. Scale bar, 100 nm. Each numbers indicates one typical synapse. (**C**) Representative synapses at 80,000X magnification as indicated in the images of (**B**). Numbers indicate the corresponding synapses shown in (**B**). Scale bar, 100 nm. (**D**) No significant differences in the density of asymmetric synapses (P = 0.6222) and length of postsynaptic density (as an estimate for the length of the active zone; P = 0.1875) were found in cerebellar molecular layer between WT and mutant mice. Error bars, mean ± SEM. n = 202 synapses from 4 WT mice and n = 439 synapses from 4 mutant mice; N.S., not significant.
