# Supplementary material for: PRRT2 deficiency induces paroxysmal kinesigenic dyskinesia by regulating synaptic transmission in cerebellum
Source: Cell Res. 2017 Oct 20;28(1):90–110. doi: 10.1038/cr.2017.128 (PMC5752836; doi:10.1038/cr.2017.128)
Supplement: Supplementary information, Figure S7 — Behavioral profile of Prrt2-mutant mouse. [file cr2017128x7.pdf]

Supplementary information, Figure S7

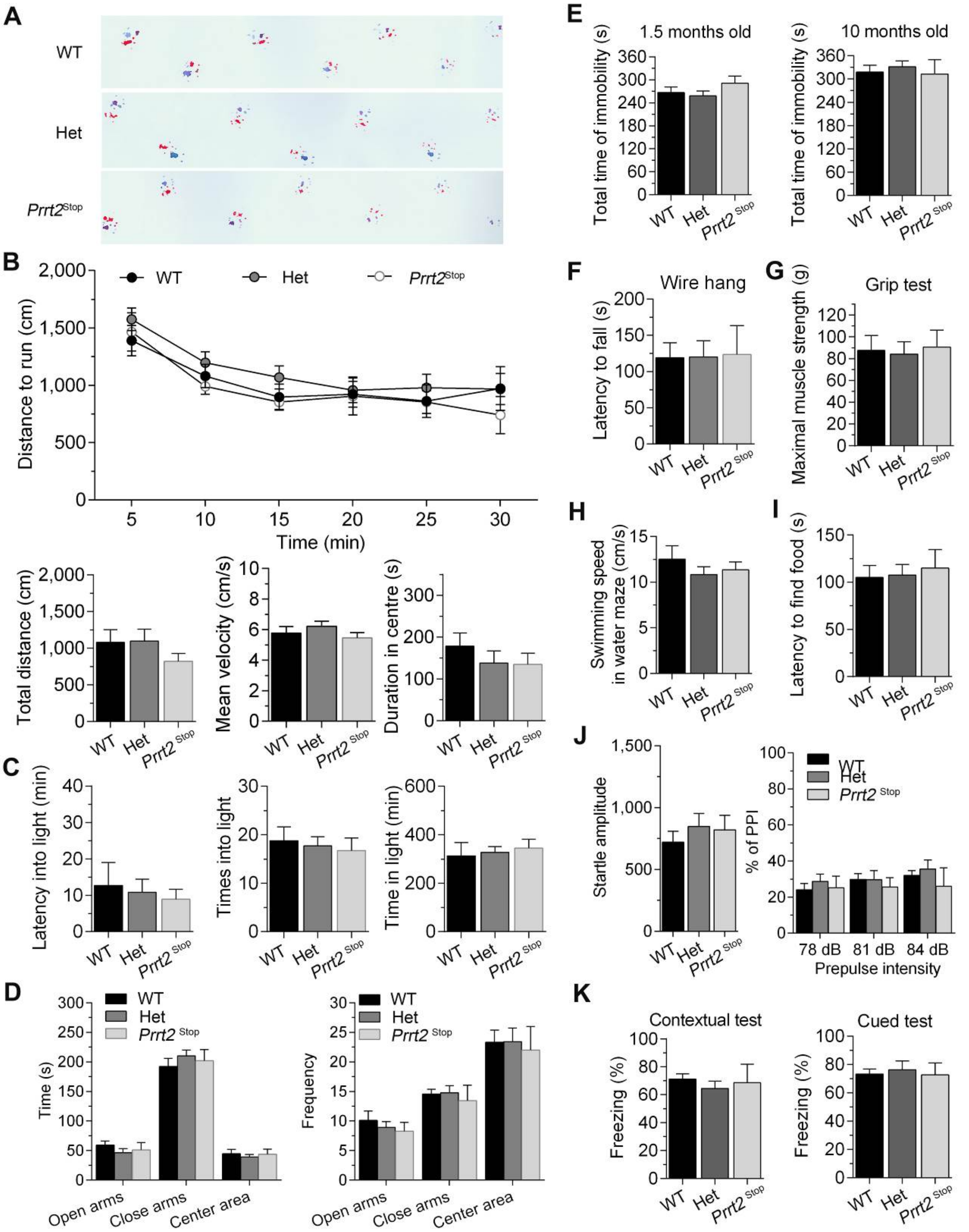

Figure S7 Behavioral profile of *Prrt2*-mutant mouse. (**A**) Gait analysis of *Prrt2*^{Stop} mice. (**B**) Open-field test. Records of the distance traveled, walking velocity, duration

in centre within 30 min in the open field showed that both homozygous and heterozygous (Het) *Prrt2*^{Stop} mice display normal locomotor activity, compared with their WT controls. (**C**) Light-dark box test. Latency of the first entry into light box, time spent in light box, and the times of entry are not altered in *Prrt2*^{Stop} mice, compared with WT mice. (**D**) Performance of mice in the elevated maze was quantified. (**E**) Tail suspension test showing that there were no differences among the groups in the total time of immobility in 6 min at the indicated ages. (**F**) Wire hang test showing that latency to fall had no difference among the mice with distinct genotypes. (**G**) Grip force measurement in *Prrt2*^{Stop}, heterozygous and WT mice. (**H**) Swimming speed in water maze was not altered in *Prrt2*^{Stop} mice. (**I**) Olfactory test. The latency to find buried food was not apparently changed in the novelty-suppressed feeding test. (**J**) Startle response and prepulse inhibition. Startle amplitude upon a 120 dB sound (left panel) and percentage of inhibition (right panel) of the original startle when receiving either 78 dB, 81 dB or 84 dB sounds prior to the 120 dB startling sound. (**K**) Short-term contextual or cued tests. Data was expressed as the percentage of time spent in freezing during the tests. For column tables, one-way ANOVA was used; while for grouped tables, two-way ANOVA was used. Error bars, mean ± SEM. $n \geqq 10, 14, 11$, for WT, heterozygous and homozygous mutants, respectively.
