# Supplementary material for: PRRT2 deficiency induces paroxysmal kinesigenic dyskinesia by regulating synaptic transmission in cerebellum
Source: Cell Res. 2017 Oct 20;28(1):90–110. doi: 10.1038/cr.2017.128 (PMC5752836; doi:10.1038/cr.2017.128)
Supplement: Supplementary information, Figure S8 — Impaired performance of Prrt2-mutant mice in beam walking and Rotarod test. [file cr2017128x8.pdf]

Supplementary information, Figure S8

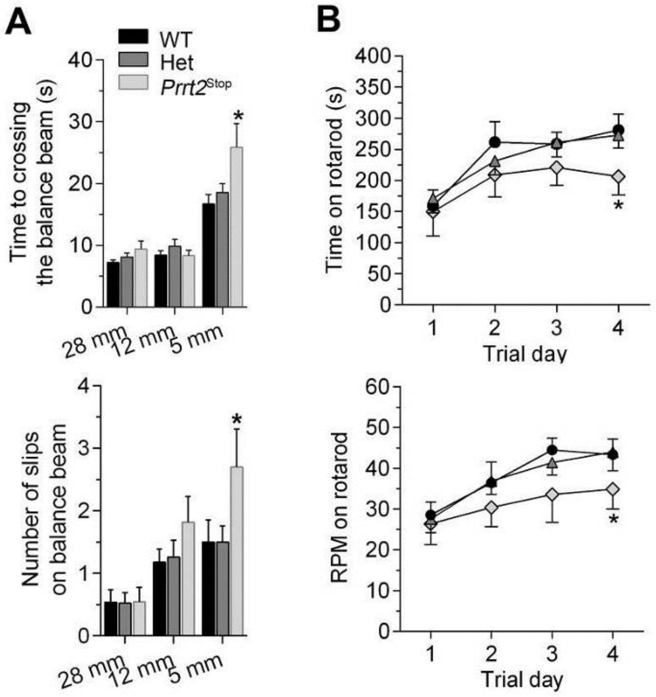

Figure S8 Impaired performance of *Prrt2*-mutant mice in beam walking and Rotarod test. (**A**) Beam balance test of *Prrt2*^{Stop} mutants and their WT littermates. (**B**) *Rota rod* test of *Prrt2*^{Stop} mutants and their WT littermates. WT, *n* = 14; heterozygous (Het), *n* = 23; homozygous *Prrt2*^{Stop}, *n* = 11; Error bars, mean ± SEM. **P* <0.05, versus WT; two-way ANOVA test.
