# Supplementary material for: PRRT2 deficiency induces paroxysmal kinesigenic dyskinesia by regulating synaptic transmission in cerebellum
Source: Cell Res. 2017 Oct 20;28(1):90–110. doi: 10.1038/cr.2017.128 (PMC5752836; doi:10.1038/cr.2017.128)
Supplement: Supplementary information, Figure S9 — Generation and verification of Nestin-Cre;Prrt2−/− mice. [file cr2017128x9.pdf]

## Supplementary information, Figure S9

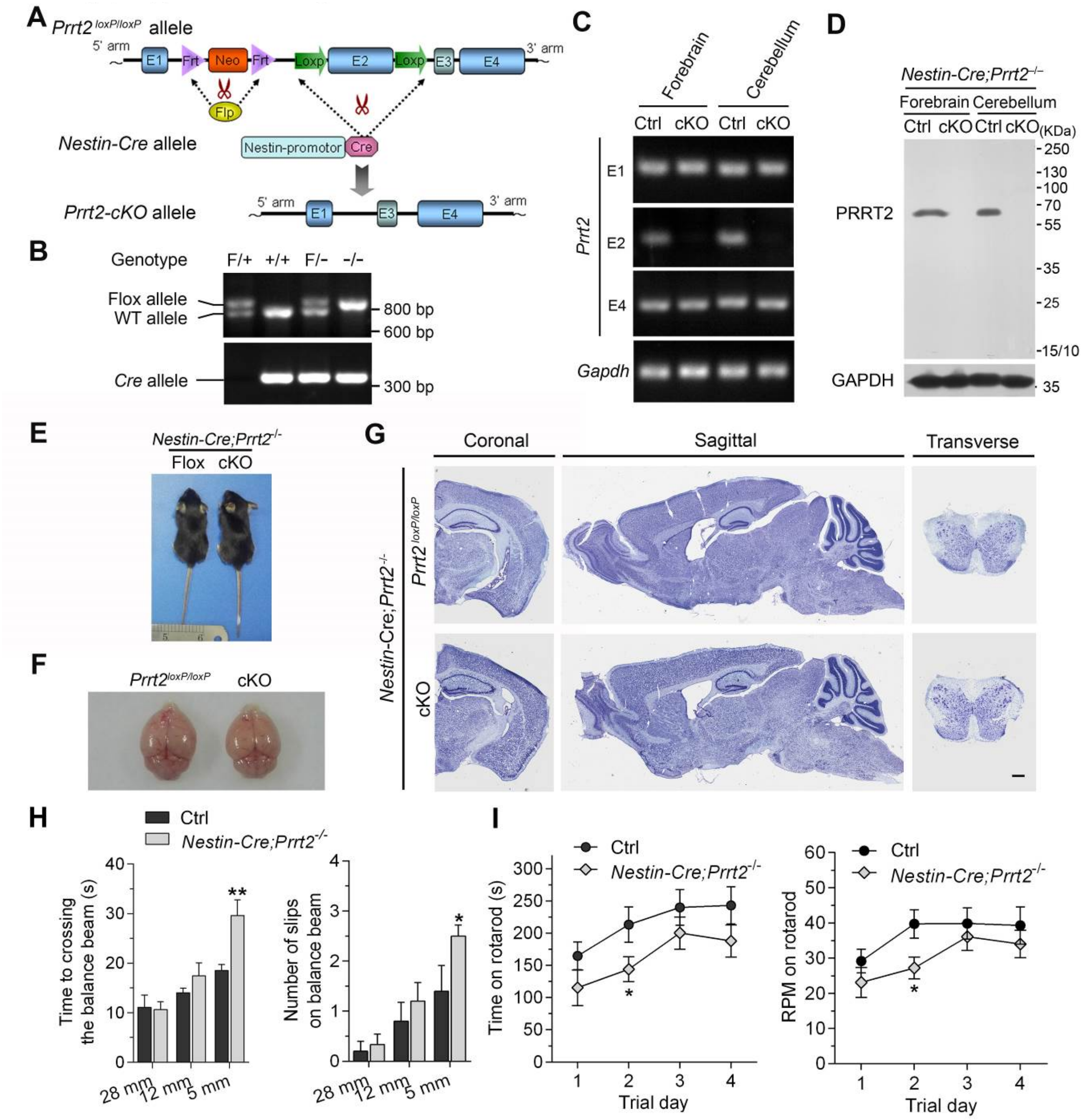


**Figure S9 Generation and verification of *Nestin-Cre;Prrt2*$^{-/-}$ mice.** (**A**) Conditional knockout of *Prrt2* in mouse CNS under the control of *Nestin-Cre*. Exon2 encoding the main part of PRRT2 was flanked by two *loxP* sequences. The *neo* cassette was ultimately deleted from the targeted alleles by *Flp* recombinase. (**B**) Representative genotyping results of the *Nestin-Cre;Prrt2*$^{-/-}$ mice. (**C**) mRNA expression of *Prrt2* in *Nestin-Cre;Prrt2*$^{-/-}$ brain was detected using probes targeting different exons as

indicated. (**D**) Western blot of expression levels of PRRT2 in brains of *Nestin-Cre;Prrt2*$^{-/-}$ mice and their control littermates. None residual PRRT2 protein was detected in *Prrt2*-knockout (KO) mouse brains. *Nestin-Cre* and *Prrt2*$^{loxP/loxP}$ mice expressed PRRT2 normally. (**E**, **F**) No obvious changes in gross morphology and brain size of the mutant mice. Error bars, mean ± SEM. (**G**) Nissl staining of sections from the mouse brains (left panel, coronal; middle panel, sagittal) and spinal cord (right panel) with the indicated genotypes showed that the gross anatomic structure of central nervous system was not apparently altered in *Nestin-Cre;Prrt2*$^{-/-}$ mice. Scale bars: 600 μm, for brain; 300 μm, for spinal cord. (**H**, **I**) Impaired performance of *Nestin-Cre;Prrt2*$^{-/-}$ mice in both beam balance test (**H**) and *Rota rod* test (**I**). Ctrl, $n$ = 10; *Nestin-Cre;Prrt2*$^{-/-}$, $n$ = 8. *Nestin-Cre* and *Prrt2*$^{loxP/loxP}$ littermates expressing PRRT2 normally were pooled as controls (Ctrl). Error bars, mean ± SEM. *$P < 0.05$ and **$P < 0.01$, versus Ctrl; two-way ANOVA test.
