# Supplementary material for: PRRT2 deficiency induces paroxysmal kinesigenic dyskinesia by regulating synaptic transmission in cerebellum
Source: Cell Res. 2017 Oct 20;28(1):90–110. doi: 10.1038/cr.2017.128 (PMC5752836; doi:10.1038/cr.2017.128)
Supplement: Supplementary information, Figure S10 — Effect of CBZ on experimentally induced dyskinesia in mouse models. [file cr2017128x10.pdf]

Supplementary information, Figure S10

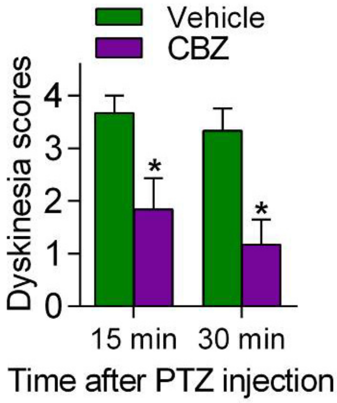

Figure S10 Effect of CBZ on experimentally induced dyskinesia in mouse models. Administration of CBZ alleviated PTZ-induced dyskinesia at the indicated time points. $n = 6$ per group. *$P < 0.05$ and ***$P < 0.001$, versus vehicle; Student's *t*-test.
