# Supplementary material for: PRRT2 deficiency induces paroxysmal kinesigenic dyskinesia by regulating synaptic transmission in cerebellum
Source: Cell Res. 2017 Oct 20;28(1):90–110. doi: 10.1038/cr.2017.128 (PMC5752836; doi:10.1038/cr.2017.128)
Supplement: Supplementary information, Figure S11 — Increased short-term facilitation of PF-PC synapses in cerebellum of GluN2C-iCre;Prrt2−/− mice. [file cr2017128x11.pdf]

Supplementary information, Figure S11

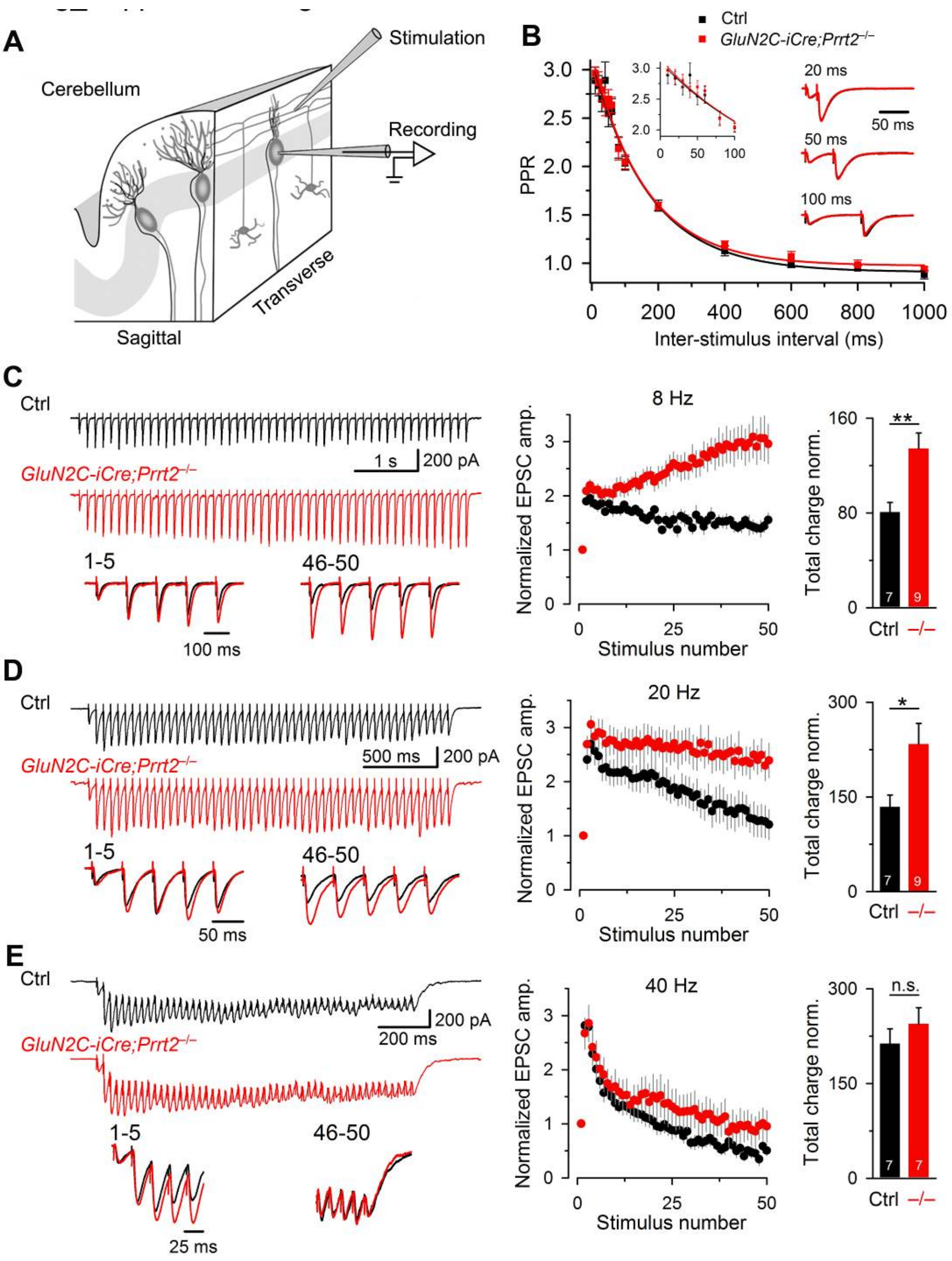

Figure S11 Increased short-term facilitation of PF-PC synapses in cerebellum of *GluN2C-iCre;Prrt2*$^{–/–}$ mice. (**A**) Illustration of the electrophysiological recording configuration. (**B**) Paired-pulse ratio (PPR) at intervals from 10 to 1000 ms in WT littermate and *GluN2C-iCre;Prrt2*$^{–/–}$ mice. Mono-exponential fits to the data were shown as solid lines. Left inset shows PPR values at very short ISIs (10-100 ms) on an expanded timescale. Right inset shows examples of paired-pulse traces at 20, 50,

100 ms intervals, scaled to the first EPSC in the pair for each interval. WT, n = 7; mutant, n = 9. (**C**-**E**) PF-EPSCs evoked by a stimulus train of 50 pulses at 8 Hz (**C**), 20 Hz (**D**), 40 Hz (**E**) in WT (black) and *GluN2C-iCre;Prrt2*$^{-/-}$ (red) mice. Left panels, representative traces of the PF-EPSCs; middle panels, normalized plot of EPSC amplitudes of each spike during trains of stimulations at the indicated frequencies; right panels, total charge transferred (normalized to that of the 1st EPSC) during the trains of 50 pulses at different frequencies. The amplitudes of evoked responses or total charge transferred were normalized to that of the 1st EPSC (WT, black; mutant, red). Case number is indicated in the bottom of each column. Error bars, mean ± SEM. $^{**}P < 0.01$ and $^{*}P < 0.05$, *versus* WT; two-sided unpaired Student's t-test.
