# Supplementary material for: PRRT2 deficiency induces paroxysmal kinesigenic dyskinesia by regulating synaptic transmission in cerebellum
Source: Cell Res. 2017 Oct 20;28(1):90–110. doi: 10.1038/cr.2017.128 (PMC5752836; doi:10.1038/cr.2017.128)
Supplement: Supplementary information, Table S1 — Sequences of PCR Primers Used in This Study [file cr2017128x19.pdf]

Supplementary information, Table S1

Table S1 Sequences of PCR Primers Used in This Study

Primer			Direction	Sequence	Accession Number
PCR	*Prrt2*	E1	Forward	CCACCTCCCTTCATTGCTCACA	NM_001102563
			Reverse	CTCAGTTGGTCCCAGTCTCATCC	
		E2	Forward	CCCCAACCTCACTCACCACC	
			Reverse	TGCCCTTCCTATTCTGTCTTCCTCA	
		E4	Forward	CCTTAGCGTCTTCACCCTCC	
			Reverse	CCAAACGGGCAGATCAATT	
		E1-E2	Forward	TTGCTCACAGACTTGTTCCCTC	
			Reverse	AGCTGCTGGCTGCCATCTT	
		E2-E2	Forward	GGTAGCCTAAGCCGTCATCC	
			Reverse	GCATAAGCGAAGGCCACAAT	
		E3-E4	Forward	TGCGTCATCAACTTAGGCG	
			Reverse	CCAGGGATAAGGTTCAGAGGATT	
	Gapdh		Forward	GGTTGTCTCCTGCGACTTCA	NM_001289726
			Reverse	CCACCACCCTGTTGCTGTAG	
Molecular Clone	*Prrt2*-shRNA-#1			GAGGAAGACAGAATAGGAA	NM_001102563
	Prrt2-shRNA-#2			GCCAGCATCCAAACCAGAT	NM_001102563
	Full-length *Prrt2*		Forward	CGAGCTCAAGCTTCGAATTCCGCC ACCATGGCAG	NM_001102563
			Reverse	TCACCATGGTGGCGACCGGTGGTT TGTCGTCATCATCCTTATAGTC	
