# Supplementary material for: PRRT2 deficiency induces paroxysmal kinesigenic dyskinesia by regulating synaptic transmission in cerebellum
Source: Cell Res. 2017 Oct 20;28(1):90–110. doi: 10.1038/cr.2017.128 (PMC5752836; doi:10.1038/cr.2017.128)
Supplement: Supplementary information, Table S2 — Commercial Antibodies Used in Western Blot and IHC Experiments. [file cr2017128x20.pdf]

Supplementary information, Table S2

Table S2 Commercial Antibodies Used in Western Blot and IHC Experiments.

Used in	Antibody	Company	Catalog Number	Titer in Use
Western Blot	Rabbit anti-PRRT2	Sigma	HPA014447-100μl	1:500
	Mouse anti-SYP	Synaptic Systems	101011	1:3000
	Mouse anti-MAP2	Neuromics	MO22116	1:1000
	Mouse anti-VGLUT 1	Synaptic Systems	135311	1:1000
	Mouse anti-GluR1	Santa Cruz	SC-55509	1:1,000
	Mouse anti-NeuN	Millipore/Chemicon	MAB377	1:1000
	Rabbit anti-GFP	Molecular Probe	A-11122	1:2,000
	Mouse anti-SYT1	Millipore	573824-200μg	1:1500
	Rabbit anti-VAMP2	Chemicon/Millipore	AB5856-50μl	1:2000
	Rabbit anti-PSD95	Cell Signaling	3450s	1:3000
	Rabbit anti-syntaxin1A	Abcam	Ab41453	1:2000
	Rabbit anti-syntaxin1B	Synaptic Systems	110403	1:500
	Mouse anti-SNAP25	Synaptic Systems	111011	1:5000
	Mouse anti-VGAT	Synaptic Systems	131011	1:1000
	Mouse anti-Flag	Sigma	F3165	1:2000
	Mouse anti-HA	Roche	11867423001	1:3000
	Mouse anti-Myc tag	Upstate/Millipore	05-724	1:3000
	Mouse anti-GAPDH	KangCheng	KC-5G5	1:5,000
	Goat anti-Actin-HRP	Santa Cruz	SC-1615	1:5,000
	Goat anti-mouse IgG-HRP	Santa Cruz	SC-2031	1:2,000
	Goat anti-Rabbit IgG-HRP	Santa Cruz	SC-2036	1:2,000
IHC/ICC	Mouse anti-MAP2	Neuromics	MO22116	1:1000
	Mouse anti-SYP	Synaptic Systems	101011	1:1,000
	Mouse anti-Cre	Chemicon/Millipore	MAB3120	1:500
	Mouse anti-SYS1	Synaptic Systems	106001	1:1,000
	Chicken Anti-GFP	Abcam	ab13970	1:3000
	Guinea pig anti-synapsin 1,2	Synaptic Systems	106004	1:200
	AlexaFluo® 488 donkey anti-rabbit IgG	Invitrogen	A-21206	1:3000
	Alexa Fluor® 488 goat anti-chicken IgG	Invitrogen	A-11039	1:3000
	Mouse anti-NeuN	Millipore/Chemicon	MAB377	1:100
	Rabbit anti-NeuN	Merck/Millipore	ABN78	1:500
	Guinea pig anti-PV	Chemicon/Millipore	AB15738	1:500
